# Re-engineering the inner surface of ferritin nanocage enables dual drug payloads for synergistic tumor therapy

**DOI:** 10.7150/thno.68459

**Published:** 2022-01-24

**Authors:** Zhuoran Wang, Yue Zhao, Shuai Zhang, Xuehui Chen, Guoming Sun, Baoli Zhang, Bing Jiang, Yili Yang, Xiyun Yan, Kelong Fan

**Affiliations:** 1 CAS Engineering Laboratory for Nanozyme, Key Laboratory of Protein and Peptide, Pharmaceutical Institute of Biophysics, Chinese Academy of Sciences, Beijing, 100101, P. R. China.; 2 University of Chinese Academy of Sciences, Beijing, 101408, P. R. China.; 3 Nanozyme Medical Center, School of Basic Medical Sciences, Zhengzhou University, Zhengzhou, 450001, P. R. China.; 4 Nanjing Nanozyme Tech Co., Ltd., Nanjing, 211500, P. R. China.; 5 China Regional Research Center, International Center for Genetic Engineering and Biotechnology, Taizhou, 225312, P. R. China.

**Keywords:** re-design of ferritin nanocage, inner surface engineering, hydrophobic drugs loading, dual drug payloads, synergistic tumor therapy

## Abstract

**Rationale:** With the advantages of tumor-targeting, pH-responsive drug releasing, and biocompatibility, ferritin nanocage emerges as a promising drug carrier. However, its wide applications were significantly hindered by the low loading efficiency of hydrophobic drugs. Herein, we redesigned the inner surface of ferritin drug carrier (ins-FDC) by fusing the C- terminus of human H ferritin (HFn) subunit with optimized hydrophobic peptides.

**Methods:** Hydrophobic and hydrophilic drugs were encapsulated into the ins-FDC through the urea-dependent disassembly/reassembly strategy and the natural drug entry channel of the protein nanocage. The morphology and drug loading/releasing abilities of the drug-loaded nanocarrier were then examined. Its tumor targeting character, system toxicity, application in synergistic therapy, and anti-tumor action were further investigated.

**Results:** After optimization, 39 hydrophobic Camptothecin and 150 hydrophilic Epirubicin were encapsulated onto one ins-FDC nanocage. The ins-FDC nanocage exhibited programed drug release pattern and increased the stability and biocompatibility of the loaded drugs. Furthermore, the ins-FDC possesses tumor targeting property due to the intrinsic CD71-binding ability of HFn. The loaded drugs may penetrate the brain blood barrier and accumulate in tumors *in vivo* more efficiently*.* As a result, the drugs loaded on ins-FDC showed reduced side effects and significantly enhanced efficacy against glioma, metastatic liver cancer, and chemo-resistant breast tumors.

**Conclusions:** The ins-FDC nanocarrier offers a promising novel means for the delivery of hydrophobic compounds in cancer treatments, especially for the combination therapies that use both hydrophobic and hydrophilic chemotherapeutics.

## Introduction

Cancer is a leading cause of mortality and a significant challenge for the healthcare system. Targeted delivery of chemotherapeutic drugs to reduce their side effects and improve their pharmacokinetics has been the focus of many investigations 1. However, the poor solubility and stability in aqueous media of most antitumor drugs significantly limited their effectiveness in chemotherapies 2, 3. The insufficient drug doses at the lesion site also led to low bioavailability after administration of these agents 4. It has been proposed that the efficacy of chemotherapy could be greatly improved by the development of a targeted drug carrier that enhances the effective solubility of hydrophobic antitumor drugs and selectively delivers them to malignant tissues, thereby increasing the effectiveness and sparing normal tissues from the cytotoxic action of the chemotherapeutic agents 1, 5.

Nanotechnology has been recognized as one of the most promising and effective strategies to improve the solubility, stability and bioavailability of hydrophobic or poorly soluble drugs 6-9. It is worth noting that nanocarriers may help overcome drug resistance of cancers through multiple mechanisms, including enhancing drug uptake and increasing drugs retention time inside tumor cells 10-14. Among the various nanoencapsulation strategies, protein nanocarrier possesses advantages in potential clinical application for being water-soluble, biocompatible, biodegradable, and non-toxic 6, 15, 16. Currently, hydrophobic drugs are usually bound to the surface of proteins or protein particles via electrostatic interactions, which may cause instability of the colloidal system. Furthermore, the loading capacity is usually relatively low, and the bound drugs are often susceptible to environmental stresses such as changes of pH, salts, and light. In contrast, hydrophobic drugs are encapsulated within the hydrophobic cores of the protein nanocarrier, which would avoid many of the issues 6. Therefore, there is an increasing demand in the field to develop novel and improved protein-based nanocarriers with high encapsulation capacity for the delivery of hydrophobic drugs, which has great potential in functional drug carrier formulations.

Among various protein nanocarriers, ferritin drug carrier (FDC), characterized by a cage-like architecture of 24 self-assembling subunits, is capable of encapsulating drugs and can be modified genetically or chemically for additional functionality, and thus has the potential to act as a cargo vehicle for effective delivery of chemotherapeutic drugs 6, 17, 18. Since its first report by Simek and Kilic in 2005 19, FDC has been further explored as a promising drug delivery vehicle. Its BBB penetration ability, self-assembly ability, symmetrical spherical architecture, pH-sensitive disassembly, and high thermal stability are particularly desirable 20-22. In addition, H-ferritin (HFn) based FDC recognizes CD71 (also known as transferrin receptor 1, TfR1), which is highly expressed on tumor cells may ensure the tumor-targeted drug delivery 17, 20, 23. It has also been demonstrated that HFn is a highly biocompatible protein carrier and stable in biological fluids, leading to markedly improvment of drug solubility and pharmacokinetics in the bloodstream 24-26.

However, the translational studies of FDC are seriously hindered by the low efficiency and low yield of drug loading process, which typically employs passive diffusion or pH dependent disassembly/reassembly methods. In the passive diffusion process, the amount of drugs loaded into HFn nanocages is usually low and mostly limited to small molecules (with size < 5 Å) 27-29. The extreme pHs (pH < 2.0 or pH > 12.0) used in pH dependent disassembly/reassembly method of loading drugs often damage the reassembly and recovery of HFn 30, 31. It has also been shown that urea dependent disassembly/reassembly of HFn nanocages may encapsulate doxorubicin (Dox), but the drug loading efficiency is still limited (∼33 Dox per HFn nanocage) 32. Interestingly, a natural thermal-response drug entry channel on the shell of HFn has been identified recently. A simple channel-based drug loading strategy was than developed, which avoided the denaturation and reassembly of HFn protein cage 33. Loading Dox into the HFn protein cage (HFn-Dox) by the channel-based method yielded higher drug loading (∼100 Dox per ferritin nanocage) and better stability than that of the denaturation-based methods 33. However, according to previous studies, most of the residues on the inner surface of mammalian ferritins are hydrophilic 33, 34. The hydrophilic nature has an electrostatic repulsive effect on hydrophobic drugs 33, and thus hinds the hydrophobic drugs to enter the ferritin nanocage. There have been several studies that use the hydrophobic 4-fold channels to load hydrophobic drugs 35-38. However, the encapsulation efficiency was limited, and heavy protein loss was found during drug encapsulation, apparently due to a partial protein aggregating in solution caused by high hydrophobicity of drugs, which greatly limited the clinical therapy 36. In addition, the loading efficiency is negatively related to the hydrophobicity of guest molecules 38. Therefore, a more efficient hydrophobic drug loading strategy is highly desired for the applications of FDC. Of note, upload of both hydrophilic and hydrophobic drugs on one FDC nanocarrier posts additional challenges.

Encapsulation of multiple drugs into the same nanocage would provide a unique opportunity to explore their synergistic anti-tumor activities 20. Compared to their free counterparts, drugs within a single nanocarrier generally exhibit higher stability, improved pharmacokinetics, better biocompatibility, befitting size distribution, and controllable release, which all contribute to the improvement of anti-tumor efficacy 10, 39, 40. In addition, this strategy shows the prospect of loading typical hydrophobic drugs that are difficult or even impossible to apply to clinics due to their high toxicity under effective therapeutic dosages, which provides the clinical potential in tumor therapy 41. It has been reported that Camptothecin (Cpt) and Epirubicin hydrochloride (Epi) acted synergistically in inhibiting cancer cell growth and preventing the development of drug resistance in animal models 39, 42. However, the clinical use of Cpt is severely limited because of its hydrophobic nature and low bioavailability 43. It is conceivable that a targeted delivery system may make Cpt a more effective chemotherapeutic drug.

**Scheme 1:**
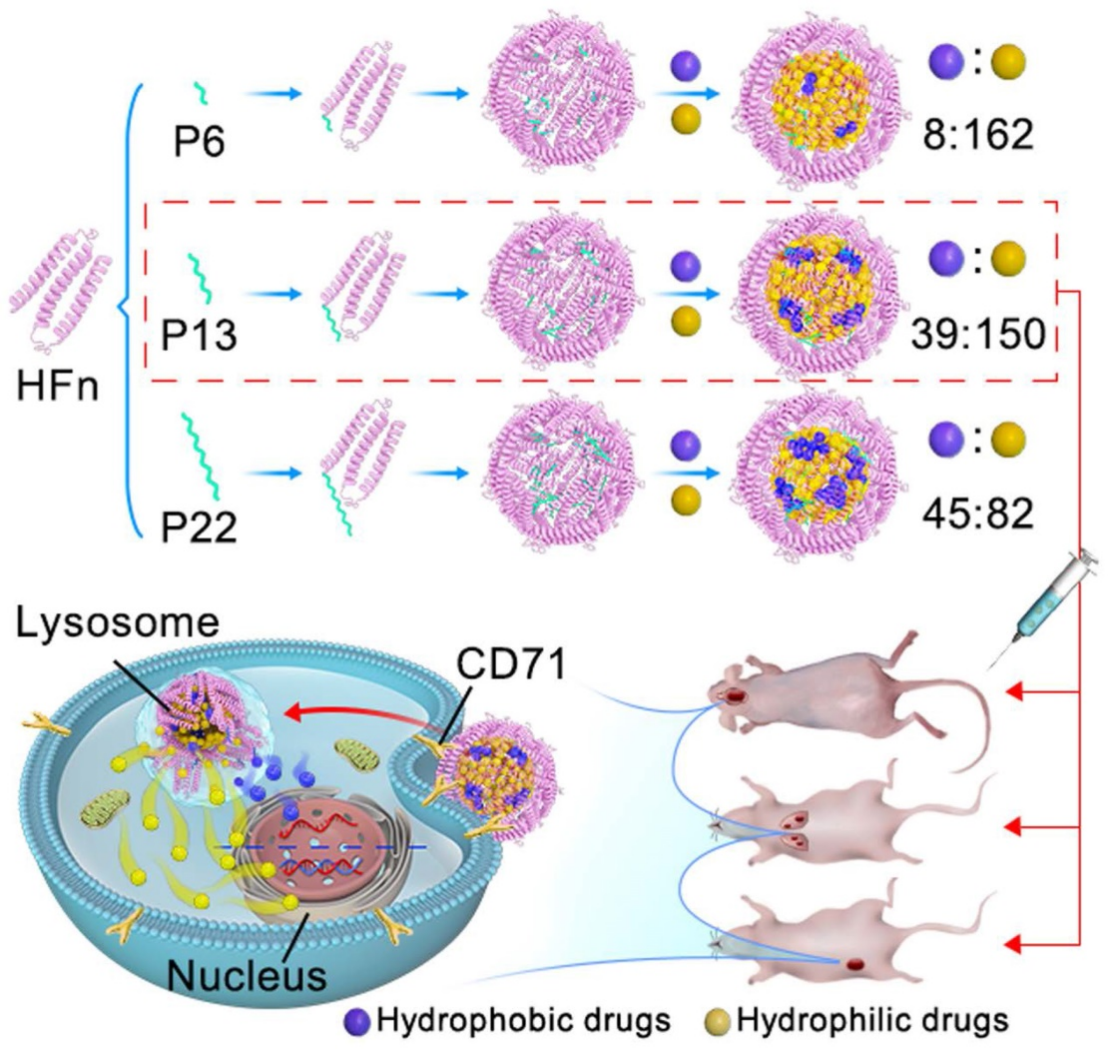
**Schematic illustration** of the key steps in Cpt/Epi@ins-FDC preparation, optimal PDRs selection, and the synergistic antitumor activity. To optimize the re-engineered nanocarriers, the ins-FDC protein cages were modified by fusing the sequence of P6, P13 or P22 to the C-terminus of the HFn to display the hydrophobic peptide motifs on the inner cavity. Cpt and Epi were co-loaded via a simple urea-thermal incubation method. Cpt/Epi@ins-FDCs P13 possessed superior drug loading capacity and exhibited synergistic cytotoxicity against glioma, metastatic liver cancer, and drug-resistant breast cancer *in vitro* and in animal models.

The purpose of this study is to design an improved FDC that is capable of loading hydrophobic and hydrophilic drugs simultaneously, which may exert anti-tumor action synergistically. We re-engineered the inner surface of ferritin nanocage by genetically-fused with a series of functional motif composed of hydrophobic peptides at the C-terminus of HFn subunit (ins-FDC), and identified the optimized ins-FDC nanocarrier by evaluating the co-loading efficiency of hydrophobic/hydrophilic drugs (**Scheme 1**). The hydrophobic drugs loading capacity of ins-FDC results from the engineered hydrophobic peptides on the inner surface of nanocage, which are located on the inner surface of 4-fold hydrophobic channels of HFn nanocage 33. The peptides form hydrophobic cores in the cavity and absorb hydrophobic drugs through hydrophobic interactions. Through the 2-fold natural drug entry channels, hydrophilic drugs are also effectively encapsulated into the nanocage 33. Thus, amphiphilic drugs can be successfully loaded with defined protein cage-to-hydrophobic/hydrophilic drugs molar ratios (PDRs). These hydrophobic peptide motifs enable the generation of a panel of amphiphilic-drug FDCs with different PDRs (ins-FDC: hydrophobic drug: hydrophilic drug) of 1:8:162 (P6), 1:39:150 (P13) and 1:45:82 (P22). By flexibly adjusting the PDR, it is possible to screen effective loading combinations based on the differences in physicochemical properties and efficacy of FDC. We have found that P13 is the most superior hydrophobic peptide motif in increasing the total drug loading capacity and enhancing synergistic cytotoxicity after co-loading Cpt and Epi. It has been shown in animal tumor models of glioma, metastatic liver cancer, and drug resistant breast cancer that Cpt/Epi@ins-FDC exhibited more effective anti-tumor actions compared to ins-FDC formulations encapsulated with Cpt or Epi alone.

Taken together, our results indicate that, in addition to their intrinsic CD71 targeting ability and programmed cascade drug release feature, Cpt/Epi@ins-FDC is stable and effective in inducing synergistic anti-tumor action. With the rapidly increasing clinical interests for hydrophobic drug carriers, we believe that the engineered ins-FDC is a promising nanocarrier for these drugs and for the combination therapies.

## Results and Discussion

### Fabrication and drug loading properties of the ins-FDC nanocarriers

#### Fabrication and self-assembly of the ins-FDC nanocarriers

**Figure 1:**
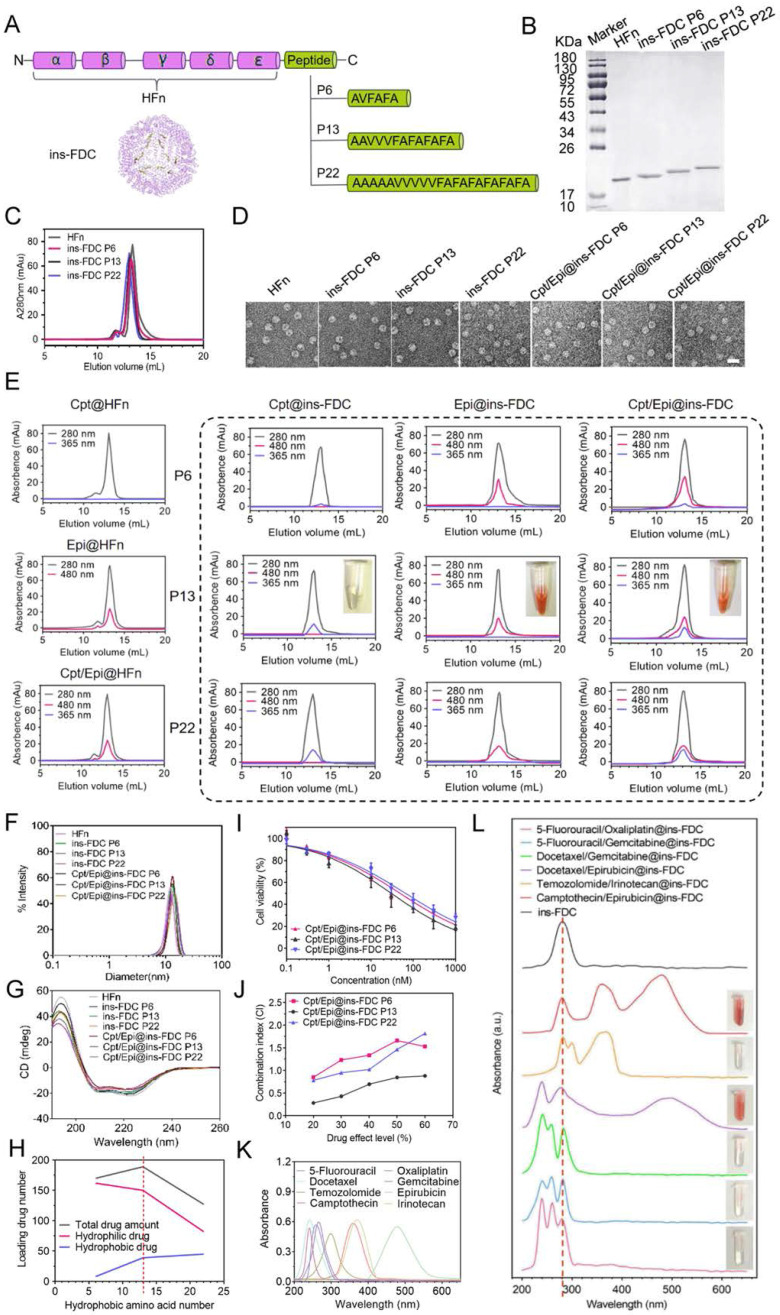
**Ins-FDC synthesis and drug loading properties.** (**A**) Schematic diagram of ins-FDC molecular design and synthesis. (**B**) SDS-PAGE characterization of HFn and ins-FDC proteins after purification. (**C**) SEC analyses of HFn and ins-FDC via in-line UV detection at 280 nm. (**D**) TEM images of HFn, ins-FDC and the drug-loaded nanocarriers. Scale bar = 20 nm. (**E**) SEC profiles of drug loaded ins-FDC nanocarriers via in-line UV detection at 280 nm (protein nanocage), 365 nm (Cpt) and 480 nm (Epi). (**F**) Hydrodynamic size of the nanocarriers measured by DLS. (**G**) CD spetrum of nanocarriers. (**H**) The relationship of hydrophobic interaction and drug loading number. (**I**) The cytotoxicity of drug-loaded ins-FDC nanocarriers. (**J**) CI of Cpt and Epi combinations via Cpt/Epi@ins-FDCs with different PDRs. The absorption spectra of various antitumor drugs (**K**) and ins-FDC loaded with different synergistic hydrophobic and hydrophilic drug combinations (**L**). The dotted line at 280 nm indicates the specific absorption peak of ins-FDC protein.

To optimize the re-engineered nanocarriers, the ins-FDC protein cages were prepared by fusing the sequence of P6, P13 and P22 to the C-terminus of the HFn subunit to display hydrophobic peptide motifs on the inner cavity 44 (**Figure 1**A). P6, P13 and P22 were composed of hydrophobic amino acid sequences of different lengths and possessed different hydrophobicity. After expression in *E. coli* and purification by anion-exchange chromatography, ins-FDC and HFn proteins were examined by using sodium dodecyl sulfate polyacrylamide gel electrophoresis (SDS-PAGE) (Figure 1B). Similar to that of unmodified HFn monomer, the hydrophobic peptides-modified FDC nanocarriers appeared as 21~23 kDa monomers in SDS-PAGE analysis (Figure 1B). It has been shown that HFn forms a self-assembly 24-subunits cage-like protein complex with an outer diameter of roughly 12 nm and an inner diameter of 8 nm 45. As shown in Figure 1C, the ins-FDC and HFn nanocages were eluted from the size exclusion chromatography (SEC) column with similar volumes, indicating that they have the same self-assembly behavior. Under transmission electron microscopy (TEM), the as-synthesized ins-FDC nanocarriers are highly monodisperse, nearly spherical in shape, and exhibit a narrow particle size distribution with a mean size of 12.47-14.04 nm (Figure 1D).

#### Ins-FDC successfully loading hydrophobic drugs

A pair of hydrophobic/hydrophilic antitumor drugs, Cpt and Epi, were selected as examples for evaluating the drug loading capacity of prepared ins-FDC nanocarriers.

Using the urea dependent disassembly/reassembly method according to our previous report 32, Cpt was absorbed into ins-FDC nanocages through hydrophobic interactions. Through the natural drug entry channel in HFn nanocage, Epi was then encapsulated into the nanocages by thermal incubation. The drug loading capacity of HFn and the engineered ins-FDC nanocarriers were analyzed by using SEC (Figure 1E). The protein nanocage, free Cpt, and Epi exhibited the typical absorption peaks at 280 nm, 365 nm, and 480 nm respectively 46. The Epi@HFn and Cpt/Epi@HFn showed absorbance peaks at 280 nm and 480 nm, indicative of the successful encapsulation of hydrophilic Epi. However, neither Cpt@HFn nor Cpt/Epi@HFn exhibited the absorption peak corresponding to Cpt, indicating that HFn cannot encapsulate the hydrophobic Cpt.

In contrast, the eluent containing Cpt/Epi@ins-FDC showed not only an absorbance peak at 280 nm, but also two specific absorption peaks at 365 nm and 480 nm, corresponding to Cpt and Epi. The nanocages Cpt@ins-FDC and Epi@ins-FDC also exhibited the absorption peak corresponding to Cpt and Epi, respectively. These results verified the successful loading of Cpt and Epi to the ins-FDC nanocarriers, and the formation of Cpt/Epi@ins-FDCs (Figure 1E), indicating that the engineered ins-FDCs overcame the obstacle in HFn and achieved the objective of effectively loading both hydrophobic and hydrophilic drugs.

The results from dynamic light scattering (DLS) analyses showed that the ins-FDC nanocarriers have a mean diameter of 13.07 ± 2.32 nm (P6), 13.38 ± 2.07 nm (P13) and 13.74 ± 2.53 nm (P22), respectively (Figure 1F). Of note, the encapsulation of the drugs did not influence the size of the nanocarriers, which is consistent with the results of TEM study (Figure 1D and Figure S1). It has been demonstrated that the main secondary structure of HFn is α-helix 47. In addition, the circular dichroism (CD) spectrum profiles of HFn and ins-FDC nanocarriers were almost the same and contained a pronounced double minimum at around 209 and 222 nm, indicating also the presence of a mainly α-helix secondary structure (Figure 1G). Thus, modification with peptides and encapsulation of drugs did not affect the overall structure of the HFn protein nanocage.

#### The hydrophobicity of the inner-surface of ins-FDC affects the pharmaceutical performance of drug loading

To evaluate the drug loading efficiency of different ins-FDC nanocarriers, we performed UV-vis spectra and SEC analyses. Based on the numbers of hydrophobic amino acid, the ins-FDC P6 was the least hydrophobic, the ins-FDC P22 was the most hydrophobic, and the ins-FDC P13 had intermediate hydrophobicity. In theory, the numbers of hydrophobic drugs loaded are correlated with the hydrophobicity of the inner-surface of ins-FDC (Figure 1E, H and Table S1). However, limited by the size of the cavity of ins-FDCs, the total drugs filled in ins-FDC P22 were less than that of ins-FDC P13 (Figure 1H and Table S1). It appears that the overlength of the hydrophobic peptide motif of P22 occupied large space of the cavity in the nanocage, which reduced the numbers of hydrophilic drugs loaded.

We first assessed the cytotoxic action of the Cpt/Epi@ins-FDCs with different PDRs on U87MG, a glioma cell line. As shown in Figure 1I and Table S2 in supporting information, the antitumor abilities of drugs-loaded ins-FDC nanocarriers were ranked as follows: Cpt/Epi@ins-FDC P13 > Cpt/Epi@ins-FDC P6 > Cpt/Epi@ins-FDC P22. For topoisomerase (topo) I and II inhibitors, the results of clinical combinations of Cpt and Epi might fall within different categories: synergism, additional or antagonistic actions 48. We used combination index (CI) -isobologram equation, a method widely used in pharmacology to study drug interactions, whose definition and calculation were described in the supporting information, to evaluate the synergistic effect of Cpt and Epi 49, 50. The Cpt/Epi@ins-FDC P13 exhibited the lowest CI value and showed synergism (CI < 1) at all the drug effect levels. In contrast, Cpt/Epi@ins-FDC P6 and Cpt/Epi@ins-FDC P22 only showed additional (CI = 1) or antagonistic (CI > 1) actions at most of the drug effect levels (Figure 1J). Thus, the dual drugs-loaded P13-modified nanocage Cpt/Epi@ins-FDC P13 exhibited synergistic anticancer activity and superior therapeutic effects, and was chosen for the further experiments.

Of note, each P13-modified ins-FDC nanocarrier can be loaded with the composition molar ratio of the ins-FDC nanocage: 39 molecules of Cpt and 150 molecules of Epi, which is the highest drug loading capacity reported for a human ferritin-based nanocarrier.

#### The optimized ins-FDC is capable of encapsulating series of hydrophobic/hydrophilic drug pairs

We further evaluated the ability of ins-FDC nanocarriers to encapsulate various clinical first-line hydrophobic/hydrophilic antitumor drug pairs. The hydrophobic/hydrophilic@ins-FDC was generated via a simple urea-thermal incubation method (Figure 1K, L). Hydrophobic@ins-FDC nanocarriers were prepared by loading hydrophobic drugs into the cavities of the ins-FDC nanocages through disassembling ins-FDC in 8 M urea in the presence of hydrophobic drugs and reassembling with gradient decreases of urea. The hydrophobic@ins-FDC nanocarriers were then thermally incubated with hydrophilic drugs to load hydrophilic drugs into the inner cavity through the natural drug entry channel. As shown in Figure 1G, H, the absorption spectra of 5-Fluorouracil/Oxaliplatin@ins-FDC, 5-Fluorouracil/Gemcitabine@ins-FDC, Docetaxel/Gemcitabine@ins-FDC, Docetaxel/Epirubicin@ins-FDC, and Temozolomide/Irinotecan@ins-FDC were all matched with the spectra of corresponding free drugs, indicatives of successful loading of these drugs pairs.

Taken together, these data demonstrated that re-engineering the inner surface of FDC with the hydrophobic peptides overcome the limitation of the hydrophilic nature of HFn based nanocages, and enables the highly efficient encapsulation of both synergistic hydrophobic and hydrophilic drugs within the nanocarrier.

Based on the above results, the ins-FDC nanocarrier re-engineered with hydrophobic peptides motif P13 on its inner cavity was selected to encapsulate hydrophobic Cpt and hydrophilic Epi, a pair of commonly used synergistic drugs, for further synergistic antitumor studies.

### Improved stability and programed drug release performance of Cpt/Epi@ins-FDC

**Figure 2:**
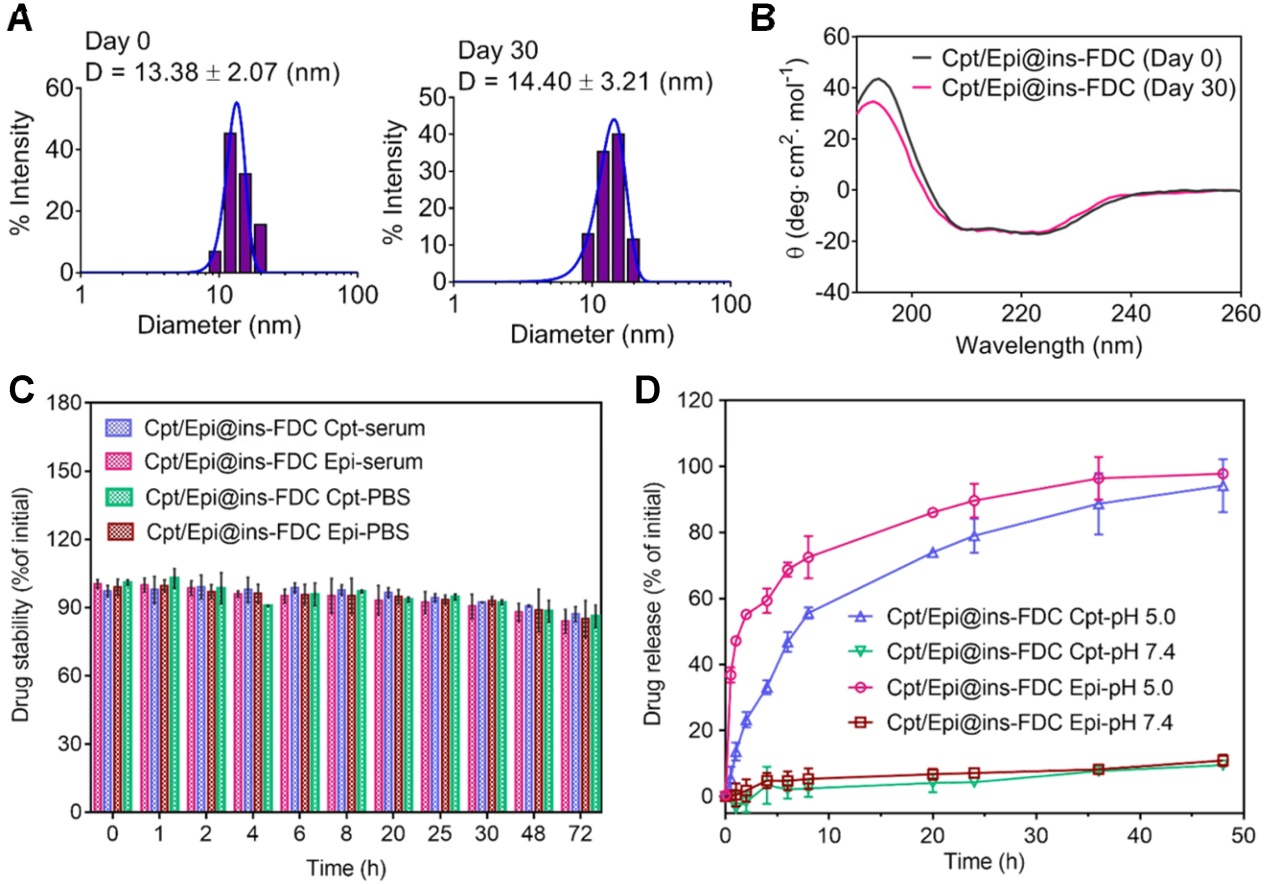
**The stability and drug release profiles of Cpt/Epi@ins-FDC.** (**A**) The hydrodynamic size distribution of Cpt/Epi@ins-FDC, as determined by DLS immediately and 30 days after the preparation. (**B**) CD spetrum of Cpt/Epi@ins-FDC. (**C**) Stability of Cpt/Epi@ins-FDC in PBS and mouse serum at 37 °C over 72 h of incubation. (**D**) Cumulative release of Cpt and Epi from the Cpt/Epi@ins-FDC in PBS at different pH values (5.0 and 7.4) *in vitro* at 37 °C. Data are shown as means ± standard deviation (SD) (n = 3).

The stability of nanocarriers in aqueous environment is important for their pharmacodynamic behaviors *in vitro* and *in vivo* 51. The stability of Cpt/Epi@ins-FDC was evaluated by determining its hydrodynamic diameter and secondary structure immediately and 30 days after its preparation. As shown in **Figure 2**A, immediately after preparation, the Cpt/Epi@ins-FDC exhibited a hydrodynamic diameter of 13.38 nm. Thirty days after preparation, the hydrodynamic diameter was 14.40 nm. Likewise, there had been little changes in the secondary structure of nanocarriers after having been stored at 4 ℃ for 30 days (Figure 2B). The Cpt/Epi@ins-FDC thus exhibited appreciable stability during a month of storage. In addition, Cpt/Epi@ins-FDC was stable under physiological conditions in mouse serum and PBS (pH=7.4). As shown in Figure 2C, there were no drug release during a 72 h dialysis. These results indicate that Cpt/Epi@ins-FDC possesses favorable stability that is beneficial for its *in vivo* pharmacodynamics.

The drug release profiles of Cpt/Epi@ins-FDC was further analyzed by dialysis in PBS of different pHs (Figure 2D). Quantitatively, less than 10% of Cpt and Epi were leaked at pH 7.4 duration a 48 h dialysis, whereas ~55.6% and ~72.5% of Cpt and Epi respectively were released in the first 8 h upon dialyzing at pH 5.0 (Figure 2D). Moreover, Epi is released relative relative rapidly due to the well-known pH-sensitive disassembling of the HFn nanocage 32, whereas, the slower release rate of Cpt was released at a slower path as the hydrophobic interaction on the inner surface was weaken gradually with the slow disintegration of the Cpt/Epi@ins-FDC nanocage. It is conceivable that the programmed and prolonged release pattern of Epi and Cpt may benefit their therapeutic effects.

### The tumor-targeted Cpt/Epi@ins-FDC exhibits programed drug release after cellular uptake

#### Cpt/Epi@ins-FDC specially targets tumor cells

**Figure 3:**
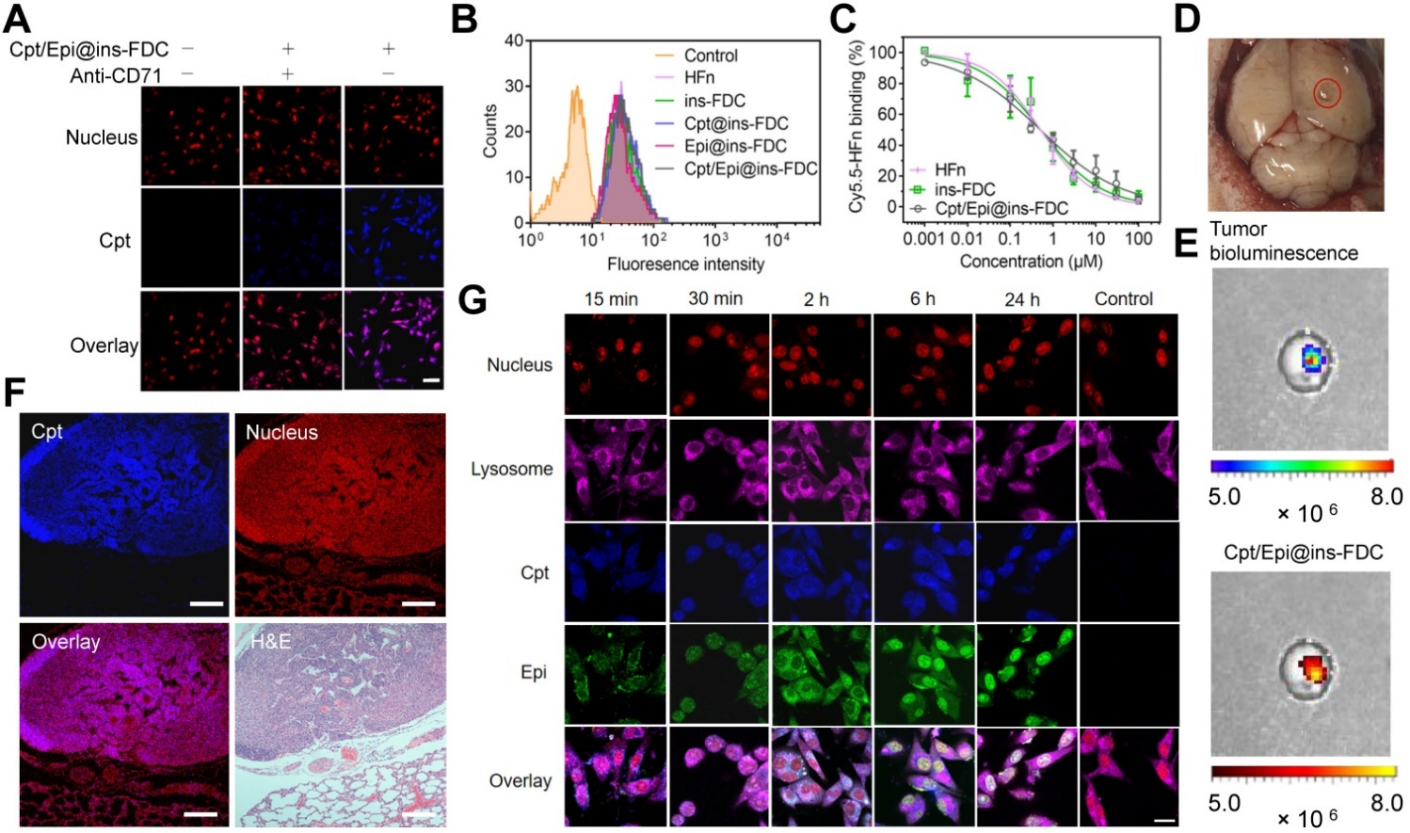
**Tumor targeting property and internalization of Cpt/Epi@ins-FDC in cells.** (**A**) Cpt/Epi@ins-FDC binds to CD71 in tumor cells. CLSM imaging of U87MG cells treated with Cpt/Epi@ins-FDC in the presence or absence of anti-CD71 mAbs. Red, propidium iodide (PI), Excitation/emission wavelength (λex/λem) = 535 nm/615 nm; blue, Cpt, λex/λem = 365 nm/500 nm. Scale bar = 20 µm. (**B**) Flow cytometry histograms of U87MG cells after incubation with different formulations for 2 h. (**C**) Competitive binding assay of Cpt/Epi@ins-FDC, ins-FDC and HFn. (**D**) Digital image of brain from U87MG tumor xenografted mouse. (**E**) Following i.v. injection of Cpt/Epi@ins-FDC, *in vivo* NIRF imaging indicated the Cpt/Epi@ins-FDC accumulated specifically in the tumor area. λex/λem = 365 nm/500 nm. (**F**) Cpt/Epi@ins-FDC based fluorescence staining, and H&E staining of paraffin-embedded lung slices from mice with lung metastasis tumors of HepG2. Red, PI, λex/λem = 535 nm/615 nm; blue, Cpt, λex/λem = 365 nm/500 nm. Scale bar = 200 µm. (**G**) Internalization of Cpt/Epi@ins-FDC by U87MG cells and the sequential released behaviour of Cpt and Epi at indicated times revealed by CLSM. Purple, Cy5 labled Lysosomal Associated Membrane Protein 1 (LAMP1), λex/λem = 650 nm/700 nm; Red, PI, λex/λem = 535 nm/615 nm; blue, Cpt, λex/λem = 365 nm/500 nm; Green, Epi, λex/λem = 485 nm/575 nm. Scale bar = 20 µm.

We examined the interaction of Cpt/Epi@ins-FDC with tumor cells using the confocal laser-scanning microscope. As shown in **Figure 3**A, pre-treatment with anti-CD71 antibody largely prevented the accumulation of Cpt in the cells, indicating that the specific binding between Cpt/Epi@ins-FDC and tumor cells is mainly mediated by CD71 (Figure 3A). Flow cytometry analyses showed that ins-FDC nanocarriers with or without loaded drugs possess similar cell binding ability to that of bare HFn (Figure 3B). The flow cytometry-based competitive binding assays demonstrated that the IC50 for Cpt/Epi@ins-FDC, ins-FDC and HFn were 0.50 ± 0.05 μM, 0.51 ± 0.10 μM, and 0.51 ± 0.08 μM respectively (Figure 3C and Table S3), confirming that drug loading did not affect the tumor cell binding ability of the HFn nanocage.

To examine the *in vivo* targeting ability of ins-FDC, an orthotopic murine glioma model was constructed by using luciferase-expressing U87MG (U87MG-LUC) cells (Figure 3D). Following intravenous (i.v.) injection of Cpt/Epi@ins-FDC, the signals of Cpt fluorescence and luciferase-catalyzed bioluminescence were largely overlapped in the tumor (Figure 3E), indicating that the ins-FDC nanocarriers penetrated BBB and specifically targeted tumor. In addition, the lungs-metastasized HepG2 nodules were specifically recognized by Cpt/Epi@ins-FDC (in purple, Figure 3F), whereas the normal lung tissues were not. These results demonstrated that Cpt/Epi@ins-FDC targets tumors specifically *in vivo*.

### Cpt/Epi@ins-FDC shows intracellular programmed drug release pattern

We further analyzed the subcellular location of the Cpt/Epi@ins-FDC in order to explore the underlying mechanisms of its internalization and intracellular drug release.

U87MG cells were incubated with Cpt/Epi@ins-FDC for 0.25, 0.5, 2, 6 and 24 h in order to analyze the cell uptake process (Figure 3G). After a 0.25 h incubation, Cpt/Epi@ins-FDC bound rapidly to the surface of tumor cells. The accumulated Cpt and Epi were detected inside the tumor cells after 0.5 h. Cpt/Epi@ins-FDC was effectively taken up by tumor cells and localized to the lysosome 2 h post the incubation. At 6h, fluorescence intensity of Epi in the nuclei was significantly stronger than that of Cpt, suggesting that Epi from the Cpt/Epi@ins-FDC was quickly released from lysosomes by the acidic environment-triggered disassambly of the nanocage. Cpt was detected in the nuclei during 6-24h, reflecting the gradually weakened hydrophobic interaction with the disintegrated ins-FDC nanocage. These results indicate that the Cpt/Epi@ins-FDC may target tumors *in vivo* and co-delivery of hydrophobic and hydrophilic drugs into tumor cells.

### Dual drug-loaded ins-FDC nanocarriers exhibit synergistic cytotoxicity

#### Cpt and Epi loaded in the ins-FDC exert synergistic cytotoxicity and kill drug resistant tumor cells

**Figure 4:**
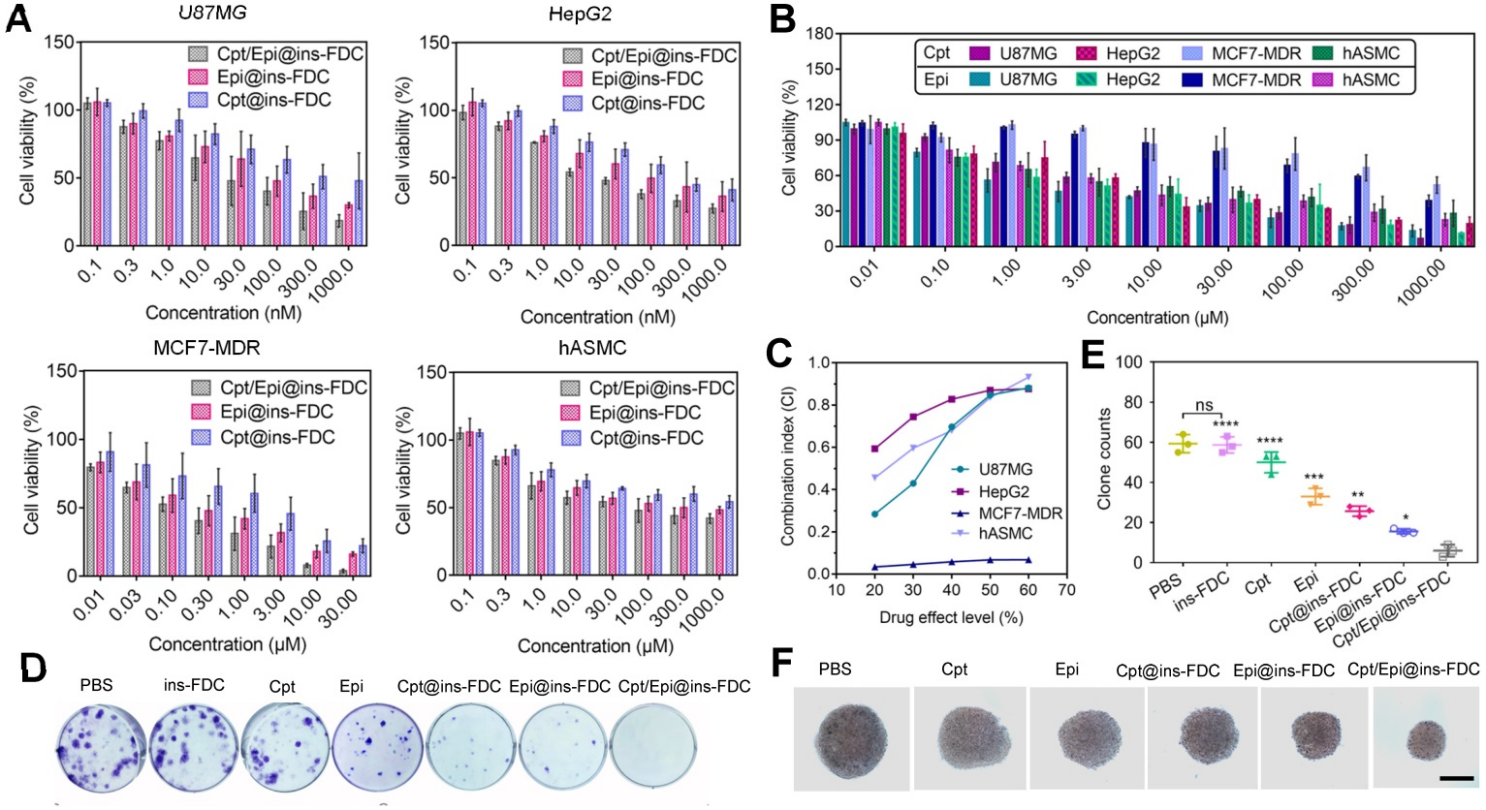
Cytotoxicity analyses of **(A)** Cpt/Epi@ins-FDC, Cpt @ins-FDC, Epi@ins-FDC and **(B)** free Cpt and Epi at different concentrations in U87MG, HepG2, MCF7-MDR and hASMC cell lines. The data represent the mean ± SD from 3 replicates for each run. **(C)** CI of Cpt and Epi combinations via Cpt/Epi@ins-FDC in different cell lines. **(D, E)** U87MG cell colonies quantification after the indicated treatments. Data are shown as means ± SD (n = 3, *p < 0.05; **p < 0.01; ***p < 0.001 and ****p < 0.0001). **(F)** Photos of MCF7-MDR cell spheroids after the different treatments on day 6. Scale bar = 20 µm.

Biocompatibility is vital for therapeutic applications of nanocarriers. The viabilities of U87MG, HepG2, MCF7-MDR cancer cells and human aortic smooth muscle cells (hASMC) treated with the ins-FDC nanocarriers were determined by using CCK8 assays. As shown in Figure S2 in supporting information, cell viabilities remained at ~95% 48h after exposed to 100,000 µM of the nanocarrier, demonstrating an excellent biocompatibility. The cell viabilities were then assessed following a 48h treatment with indicated concentrations of Cpt/Epi@ins-FDC, Cpt@ins-FDC, and Epi@ins-FDC. Cpt/Epi@ins-FDC exhibited significantly enhanced cytotoxicity compared with single-drug loaded formulations (Cpt@ins-FDC and Epi@ins-FDC) (Figure 4A and Table S4). The enhanced action of the dual-loaded ins-FDC might be attributed to the synergistic effect of Cpt and Epi combinations as well as the programmed release of drugs. Of note, the drugs-loaded nanocarriers also kill effectively the multiple drug-resistant MCF7-MDR cells, likely as a result of its unique drug delivering and releasing manners (Figure 4A, B). As shown in Figure 4C, the CI values of Cpt/Epi@ins-FDC were less than 1 at the effect drug levels, indicating a strong synergistism between nanocarrier-loaded Cpt and Epi. We also analyzed the cytotoxicity of Cpt/Epi@ins-FDC on hASMC, which expresses low level of CD71. It was found that the IC50 of Cpt/Epi@ins-FDC on hASMC cells was significantly higher than those U87MG and HepG2 cells, supporting the important role of CD71 in conferring the enhanced cytotoxic action of Cpt/Epi@ins-FDC (Figure 4A and Table S4).

It is worth noting that ins-FDC loaded with other pairs of hydrophobic and hydrophilic anticancer drugs also achieved markedly improved cancer cell killing than that of hydrophobic@ins-FDC and hydrophilic@ins-FDC (Figure S3 and Table S5), indicating that the ins-FDC possesses broad-spectrum therapeutic application prospects.

#### The effects of Cpt/Epi@ins-FDC on clonogenicity and 3D cell spheroids

We also evaluated the effects of ins-FDC loaded with Cpt and/or Epi on U87MG tumor cells colony formation. As shown in Figure 4D and E, tumor cells treaed with dual drug-loaded Cpt/Epi@ins-FDC generated a few progeny colonies (average 6 colonies), while these exposed to ins-FDC alone produced about 10 times more colonies (average 59 colonies). Moreover, free Cpt and Epi exhibited only minimal inhibitory activities on colony formation (average 50 and 33 colonies), and ins-FDC loaded with Cpt or Epi alone had intermediate inhibitory effects (average 25 and 18 colonies).

We also generated 3D spheroids from MCF7-MDR cells to evaluate the therapeutic effect of Cpt/Epi@ins-FDC. While the volume of the spheroids decreased significantly after treated with Cpt/Epi@ins-FDC, free Cpt and Epi only reduced the spheroids slightly compared with the vehicle (Figure 4F and Figure S4), and ins-FDC loaded with Cpt or Epi alone had intermediate inhibitory effects.

Taken together, these results demonstrated that the drugs-loaded ins-FDC may exert effective and synergistic antitumor action in multiple system, including the chemo-resistant 3D cultured tumor cells.

### Systemic biodistribution and plasma pharmacokinetics

**Figure 5:**
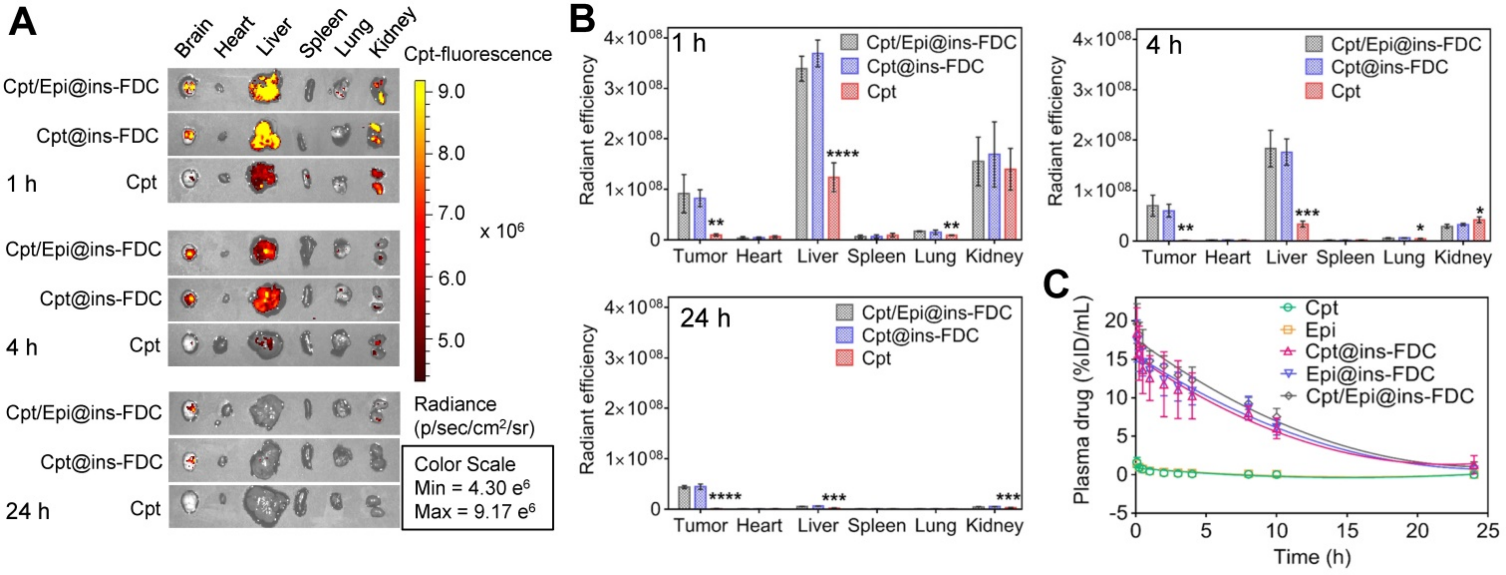
***In vivo* biodistribution and plasma pharmacokinetics of Cpt/Epi@ins-FDC.** (**A**) NIRF images of the major tissues at 1, 4 and 24 h post-injection. λex/λem = 365 nm/500 nm. (**B**) Quantification of the fluorescence in tumor and the major tissues at 1, 4 and 24 h post-injection. Free Cpt treated groups were compared with these treated with nanocarrier groups. Values are means ± SD for 3 mice. *p < 0.05; **p < 0.01; ***p < 0.001 and ****p < 0.0001. (**C**) Plasma concentrations of drugs at indicated times postinjection (n = 3).

To assess the biodistribution of our ins-FDC nanocarriers *in vivo*, we injected Cpt/Epi@ins-FDC into the tail vein of U87MG glioma-bearing mice and monitored the drug distribution by fluorescence imaging. The *ex vivo* imaging of tumors show that injection of Cpt/Epi@ins-FDC or Cpt@ins-FDC led to markedly stronger fluorescence than that of free Cpt after 1h (**Figure 5**A). A strong intratumoral signal remained 4h after injection of Cpt/Epi@ins-FDC or Cpt@ins-FDC, but not that received free Cpt. Examining its systemic biodistribution found that Cpt/Epi@ins-FDC was accumulated in the liver and kidney during the first hour post-injection, as indicated by the intense fluorescence signal in these organs. Cpt/Epi@ins-FDC was rapidly cleared after 4 h, and the signal was barely detectable 24 h later (Figures 5B). We compared our results with that of a published non-functionalized HFn 25, 32 and found very similar biodistribution and clearance kinetics, suggesting that the functionalization with FDC moiety does not alter the overall biodistribution and clearance of the nanocarriers.

The pharmacokinetic behaviors of the Cpt and Epi were also examined. The serum drug concentrations at 0-24h post administration and the pharmacokinetic parameters were shown in Figure 5C and Table S6. Cpt/Epi@ins-FDC showed an apparent blood half-life of 7.20 ± 0.14 h, which was 12.4 and 10.3 times longer than that of free Cpt (0.58 ± 0.13 h) and Epi (0.70 ± 0.16 h) respectively. The area under the curve (AUC) of Cpt/Epi@ins-FDC (177.95 ± 19.75 %ID mL^-1^ h) was 95.2 and 90.3-fold of free Cpt (1.87 ± 0.87 %ID mL^-1^ h) and Epi (1.97 ± 0.55 %ID mL^-1^ h). The significantly longer plasma half-life and higher AUC of ins-FDC apparently improved the drug retention in the systemic circulation and facilitate the time-dependent drug accumulation in tumors.

### Improved maximum tolerated dose (MTD) and biosafety of Cpt/Epi@ins-FDC

**Figure 6:**
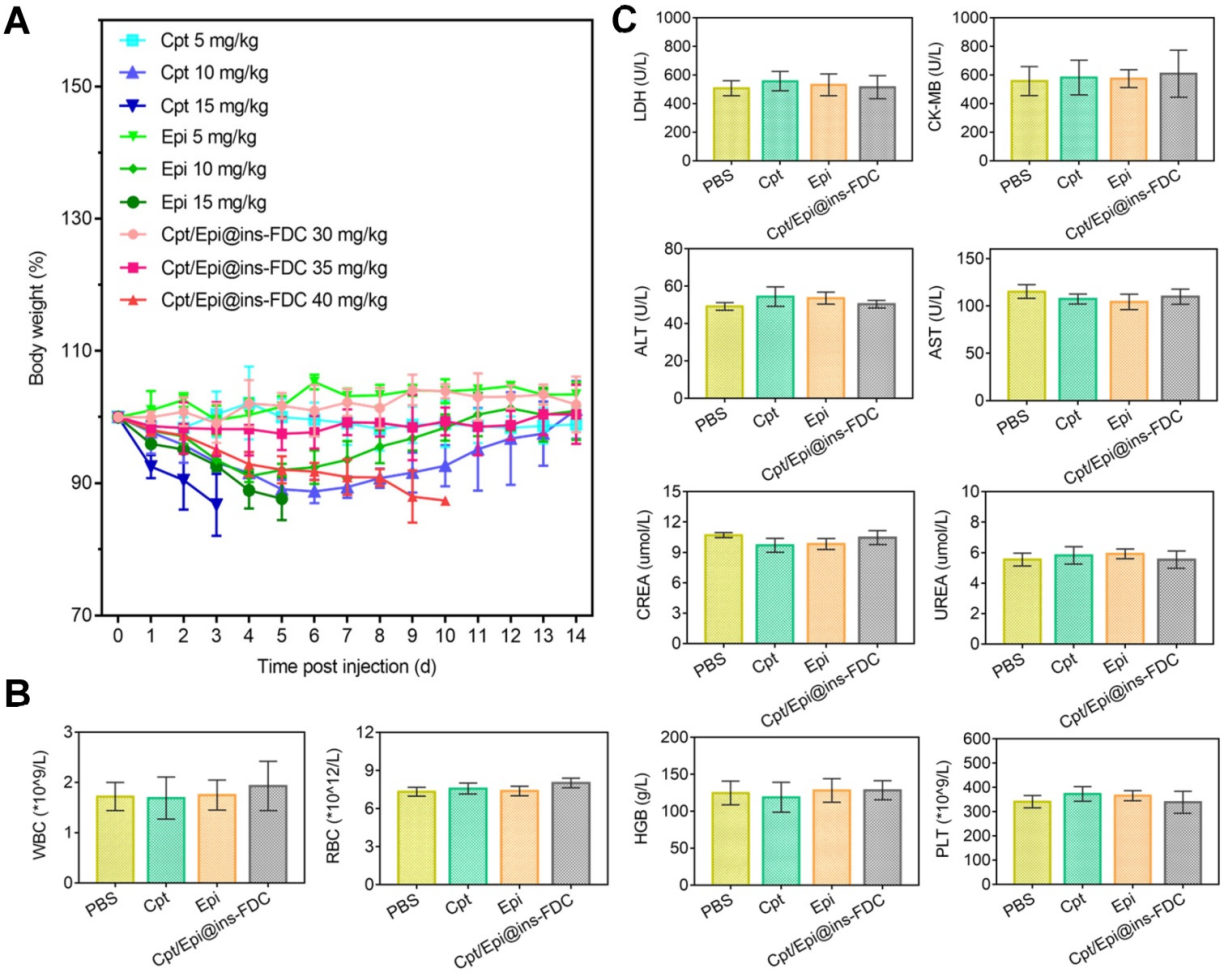
** (A)** MTD study of Cpt/Epi@ins-FDC, free Cpt and Epi. Healthy female BALB/c nude mice (n = 3) were administered i.v. on day 0 with different concentrations of PBS, free Cpt (5, 10, or 15 mg kg^-1^), free Epi (5, 10, or 15 mg kg^-1^), or Cpt/Epi@ins-FDC (30, 35, or 40 mg kg^-1^). **(B)** Blood routine data of different treatments, including WBC, RBC, HGB and PLT. **(C)** Serum biochemistry data reflecting heart function including LDH and CK-MB, liver function including ALT and AST, kidney function including CREA and UREA. Data are expressed as means ± SD (n = 3).

To assess its toxicity, healthy mice were injected with 30, 35 and 40 mg kg^-1^ of Cpt/Epi@ins-FDC at a high dose (30, 35 and 40 mg kg^-1^) and monitored for 14 days (**Figure 6**A). No significant body weight loss was found in mice received 30 and 35 mg kg^-1^ of Cpt/Epi@ins-FDC. Whereas mice given 40 mg kg^-1^ of Cpt/Epi@ins-FDC showed gradually loss of body weight, which decreased to below 85% of the initial weight in 6 days. Thus, the MTD of Cpt/Epi@ins-FDC is close to 35 mg kg^-1^, which is 5-10 folds higher than that of free Cpt and Epi (10 mg kg^-1^).

To evaluate its biosafety, blood was collected from mice 15 days post injection of Cpt/Epi@ins-FDC at 35 mg kg^-1^, and free Cpt or Epi at 10 mg kg^-1^ (Figure 6B, C). We quantified parameters associated with heart, liver and kidney functions. The levels of LDH, ALT, AST, CK-MB, CREA, HGB, PLT and UREA as well as WBC and RBC were all within normal ranges. These results indicate that the Cpt/Epi@ins-FDC does not induce apparent systemic toxicities.

### Synergistic antitumor efficacy

To demonstrate the feasibility of Cpt/Epi@ins-FDC for tumor therapy *in vivo*, U87 glioma tumors, hepatocellular carcinoma HepG2 lung metastases nodules, and MCF7/MDR drug-resistant breast tumor, which are refractory to chemotherapies for different mechanisms 52-54, were chosen as models.

#### Effective suppression of tumors

**Figure 7:**
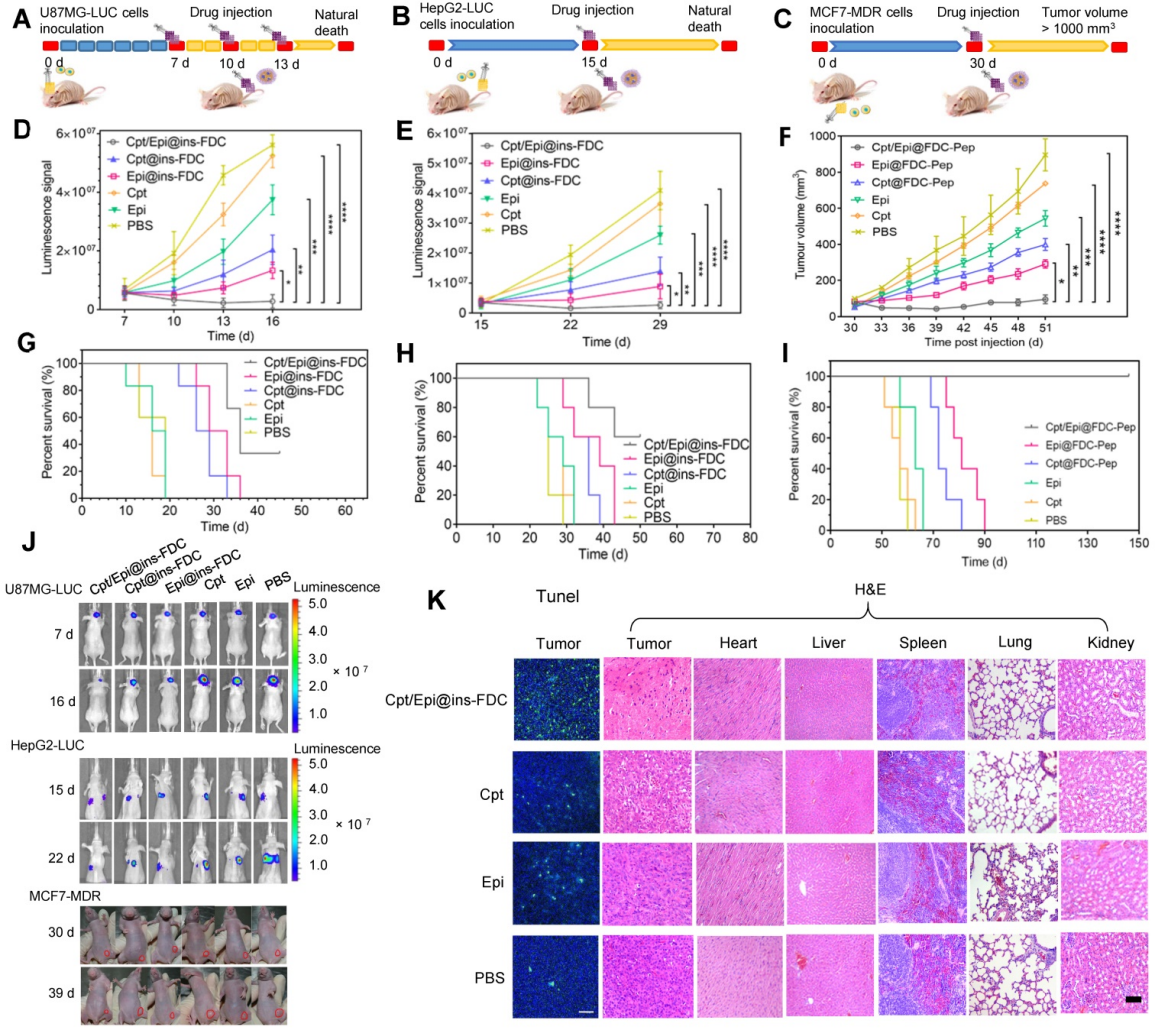
**Enhanced tumor therapeutic efficiency of Cpt/Epi@ins-FDC in U87MG-LUC, HepG2-LUC and MCF7-MDR bearing mice. (A-C)** Schematic of the established tumor models and therapeutic treatments. **(D-F)** The U87MG-LUC, HepG2-LUC and MCF7-MDR tumor growth curves after treating with either Cpt/Epi@ins-FDC, Cpt@ins-FDC, Epi@ins-FDC, Cpt, Epi, or PBS. Treatments were performed on day 7, 10, and 13 for U87MG-LUC, day 15 for HepG2-LUC and day 30 for MCF7-MDR tumor bearing mice. Data are shown as means ± SD (n = 5-6; *p < 0.05, **p < 0.01, ****p < 0.0001). **(G-I)** Survival curves in different treatment groups. **(J)** *In vivo* bioluminescence images of U87MG-LUC, HepG2-LUC and digital pictures of MCF7-MDR bearing mice that were i.v. injected with different formulations. **(K)** Representative photomicrographs of TUNEL assay and H&E stained sections.

Next, we assessed the overall therapeutic effects of Cpt/Epi@ins-FDC in animal tumor models made from U87MG-LUC glioma, HepG2-LUC lung-metastasized liver cancer, and MCF7-MDR drug-resistant breast cancer cells in comparison with Cpt@ins-FDC, Epi@ins-FDC, free Cpt and Epi. In the U87MG-LUC glioma model (Figure 7A), Cpt/Epi@ins-FDC and indicated agents were injected intravenously at day 7, 10, and 13 post tumor inoculation. For the treatment of HepG2-LUC and MCF7-MDR-derived mouse models, the therapeutic effects and toxicity of various agents were evaluated by a single injection of MTD dose at day 15 and 30 respectively (Figure 7B, C).

Compared to control group received PBS, tumors in mice given free Cpt or Epi reduced growth moderately, and tumors in mice administrated with Cpt@ins-FDC or Epi@ins-FDC had marked reduction in growth. Of note, tumors in mice treated with Cpt/Epi@ins-FDC cease to enlarge completely (Figure 7D-F and Figure S5). In H&E stained lung sections of HepG2-LUC tumor models, the numbers of metastatic nodules in the Cpt/Epi@ins-FDC-treated group were significantly less than these of the other groups (Figure S6). The regression of tumor growth also correlated well with the increase of animal survival (Figure 7G-J) and ceasing of body weight loss (Figure S7). Treatment with Cpt/Epi@ins-FDC resulted in a median survival time of 36 days for mice with U87MG-LUC glioma, 60% survival of mice with HepG2-LUC tumors for up to 50 days, and 100% survival of mice bearing MCF7-MDR tumors for up to 150 days. These were significant improvements over free drugs, and single-drug loaded Cpt@ins-FDC and Epi@ins-FDC.

#### Cpt/Epi@ins-FDC promotes tumor cell apoptosis without biotoxicity to normal tissues

The anti-glioma actions of various treatments were further evaluated by the TUNEL assay. Cpt/Epi@ins-FDC induced significantly more apoptotic cell death compared to Cpt@ins-FDC and Epi@ins-FDC. Only a few apoptotic cells were present in glioma tissues from mice given free Cpt or Epi alone. (Figure 7K and Figure S8). Histopathological examination revealed that Cpt/Epi@ins-FDC treatment resulted in loss of cell architecture in tumor sections, whereas no tissue damages were found in other organs examined (Figure 7K). These results demonstrated the superior capability of Cpt/Epi@ins-FDC in killing tumor *in vivo* without significant side effects to normal tissues.

## Conclusion

To develop FDC as a drug delivery platform encapsulating hydrophobic drugs, we have re-engineered the inner surface of the FDC nanocage by genetically fusing hydrophobic peptides with the C-terminus of HFn. The hydrophobic peptide was displayed on the inner surfaces of ins-FDC, increased the absorption of insoluble drugs, and also conferred protection to some specific drug molecules by entrapment them in the ins-FDC protein nanocage. The drug loading ability and cytotoxicity of the modified ins-FDC could also be regulated via changing the lengths of the peptide to modify its hydrophobicity.

This paper reports, for the first time, the possibility to entrap hydrophobic and hydrophilic antitumor drugs within HFn nanocage using a simple urea-thermal incubation technique. Importantly, the urea-thermal incubation loading method maximizes the loading capacity of hydrophilic and hydrophobic drugs at the same time and does not affect their further functional properties (stability, receptor affinity, payload release kinetics, etc.). We believe that these results laid a solid foundation for future studies focused on the encapsulation of hydrophobic molecules into ferritin.

The novel construct showed hydrophobic drug inclusion capacity, targeted drug delivery and post loading particle stability, and significant half-life extension. The drugs loaded in the inner cage of ins-FDC were detected to show the Epi-Cpt programed release. In addition, dual-drug loaded Cpt/Epi@ins-FDC exhibited a higher synergistic antitumor effect than the single-drug loaded ones and efficiently overcomes the tumor cell drug resistance. Moreover, it was demonstrated in our study that hydrophobic peptides-functionalized ins-FDC is a highly promising tumor cell-selective drug delivery system to encapsulate various clinical first-line hydrophobic/hydrophilic antitumor drug pairs. In addition to being used for tumor treatment, it is also expected to be used in a variety of biomedicine fields such as biocatalysis and molecular diagnosis to provide more effective synergistic treatment strategies for diseases. Overall, the newly synthesized ins-FDC nanocarrier is a safe and efficient drug delivery system with potential application in hydrophobic drug delivery and efficient synergistic therapy, holding great potential for further clinical translation in biomedical field.

## Experimental Section

### Preparation of hydrophobic and hydrophilic drug-loaded ins-FDC nanocarriers

The design of ins-FDC nanocarriers were constructed by linking the sequence of P6, P13 and P22 (AVFAFA, AAVVVFAFAFAFA, and AAAAAVVVVVFAFAFAFAFAFA) to the C-terminus of HFn through a flexible amino acid sequence GGSG, to display peptides on the inner cavity. The recombinant plasmid ins-FDC-pET-22b (+) was expressed in *E. coli.* Transetta (DE3) (TransGen Biotech) and purified by SEC (Amersham Pharmacia Biotech). The purification process of proteins was monitored with SDS-PAGE.

The Cpt/Epi@ins-FDC and Cpt/Epi@HFn were prepared via a simple urea-thermal incubation method. Cpt@ins-FDC and Cpt@HFn were prepared by loading Cpt into the cavities of ins-FDC and HFn nanocages through disassembling ins-FDC and HFn in 8 M urea in the presence of Cpt, followed by a reassembling process with a series of stepwise gradients of urea from 7 M to 0 M. The Cpt@ins-FDC and Cpt@HFn were then thermally incubated with Epi to encapsulate hydrophilic Epi into the inner cavity through the natural drug entry channels. The free drug molecules were removed using a dialysis bag (MWCO 3500 Da, Thermofisher Scientific). 5-Fluorouracil/Oxaliplatin@ins-FDC, 5-Fluorouracil/Gemcitabine@ins-FDC, Docetaxel/Gemcitabine@ins-FDC, Docetaxel/Epirubicin@ins-FDC, and Temozolomide/Irinotecan@ins-FDC were prepared by the same method as described above.

### Characterizations

The self-assambly of ins-FDC and successful loading of drugs were demonstrated on a Superdex 200 10/300 GL column (GE Healthcare) connected to a SEC system, using the in-line UV detection at 280 nm (HFn protein cage) , 365 nm (Cpt) and 480 nm (Epi). The amounts of drugs were measured by UV-vis spectra and the concentration of ins-FDC was determined by a bicinchoninic acid assay (BCA assay) kit (Sigma). The drug loading efficiency, cytotoxicity and Combination Index (CI) of ins-FDCs to tumor cells were assessed to identify the optimal hydrophobicity peptide motif for further study. DLS was carried out to measure the particle size of nanocarriers. And the morphology was characterized by TEM. The secondary structure of ins-FDC nanocarriers were analysised by CD.

### *In vitro* drug release and stability study

10 mg Cpt/Epi@ins-FDC samples in 2 mL PBS solution were transferred to dialysis bags that was directly placed into 30 mL of buffer solution at pH values of 7.4 and 5.0 at 37 °C. The concentrations of the released drugs were calculated by the detection of the fluorescence intensity. The stability evaluation was performed with the same method by incubating Cpt/Epi@ins-FDC with PBS (pH =7.4) or normal mouse serum.

### Targeting ability and cell-binding assays

To investigate the receptor mediated targeting of Cpt/Epi@ins-FDC interacted with CD71, Cpt/Epi@ins-FDC (10 µM) was added into U87MG cells for 2 h after pre-incubated with or without anti-CD71. Cells nuclei were stained by propidium iodide (PI) (λex/λem = 535 nm/615 nm). Fluorescence intensity of Cpt (λex/λem = 365 nm/500 nm) was measured by CLSM (Carl Zeiss LSM 700) and quantified by Image J software.

Ins-FDC and HFn were labeled with Cy5 according to the manufacturer's protocol, and the Cy5-ins-FDC was further loaded with Cpt or Epi by the same procedure described previously. For the binding analysis, 100 μL detached U87MG cell suspensions (2.5 × 10^6^ cells mL^-1^) were incubated with 10 μM of Cy5-conjugated Cpt/Epi@ins-FDC, Cpt@ins-FDC, Epi@ins-FDC, ins-FDC, HFn or PBS (45 min at 4 °C). Cells were analyzed by FACS. Competitive binding assay was further performed by using FACS. U87MG cells were incubated with Cy5-labeled HFn in the presence of increasing concentrations of unlabeled Cpt/Epi@ins-FDC, ins-FDC or HFn. The fluorescence of Cy5 was evaluated to quantitatively compare the binding activity of Cpt/Epi@ins-FDC, ins-FDC and HFn to CD71.

### Intracellular trafficking of Cpt/Epi@ins-FDC

U87MG cells were cultured and incubated with Cpt/Epi@ins-FDC at 37 °C for 0.25, 0.5, 2, 6 and 24 h. Lysosomes were stained with Cy5 labled Lysosomal Associated Membrane Protein 1 (LAMP1), and the cell nuclei were further stained by PI. Samples were analyzed under a CLSM. λex/λem = 650 nm/700 nm for Cy5; λex/λem = 535 nm/615 nm for PI; λex/λem = 365 nm/500 nm for Cpt; and λex/λem = 485 nm/575 nm for Epi.

### *In vitro* cytotoxicity

The cytotoxicities of Cpt/Epi@ins-FDC P6, Cpt/Epi@ins-FDC P13, and Cpt/Epi@ins-FDC P22 on U87MG cells were determined using CCK8 assay. The half-maximal inhibitory concentration (IC50) was calculated based on the protein concentration as previously reported 55 using the GraphPad Prism 7.0 software (GraphPad Software, CA, USA). The Combination Index (CI) was measured according to the Chou and Talalay's method (CI > 1 represents antagonism, CI = 1 represents additive and CI < 1 represents synergism) 56. The *in vitro* cytotoxicity of Cpt/Epi@ins-FDC, Cpt@ins-FDC, Epi@ins-FDC, free Cpt, free Epi, and ins-FDC against U87MG, HepG2 and MCF7-MDR tumor cell lines and hASMC cells were compared.

### Cell colony formation assay

2 × 10^3^ of U87MG cells were seeded into 6-well plates and incubated with drug-loaded ins-FDC nanocarriers at 37 °C for 24 h. Then the drug solution was replaced by fresh culture medium. Crystal violet solution was applied to investigate cell colonies on day 10 when macroscopic cell colonies were formed.

### *In vitro* therapeutic effect of Cpt/Epi@ins-FDC

2 × 10^3^ MCF7-MDR cells were seeded into ultra-low attachment round bottom 96-well plates (Corning, America) to form cell spheroids. After Cpt/Epi@ins-FDC, Cpt@ins-FDC, Epi@ins-FDC, free Cpt, free Epi, and PBS was added for 8 h incubation, the images and diameters of the cell spheroids were recorded for 6 days. The volume of the spheroids was calculated according to formula:

V = 4/3×π× (d/2)^2^ (1)

### *In vivo* biodistribution and pharmacokinetics

All animal experiments were performed with the approval of the Institutional Animal Care and Use Committee at the Institute of Biophysics, Chinese Academy of Sciences (SYXK2019021). For biodistribution study, Cpt/Epi@ins-FDC, Cpt@ins-FDC and free Cpt were injected to U87MG-LUC glioma-bearing BALB/c-nu mice (6-week-old) with a dose of 3 mg kg^-1^ Cpt equivalents. After 1, 4, and 24 h of injection, the tumors as well as major organs were collected and imaged with the IVIS Lumina II imaging system.

To determine the pharmacokinetics, Cpt/Epi@ins-FDC, Cpt@ins-FDC, Epi@ins-FDC, free Cpt and Epi in equivalent drug doses of 1.35 mg kg^-1^ Cpt and 8.65 mg kg^-1^ Epi was i.v. injected into BALB/c nude mice (n = 5). At different times after injection blood was collected and the plasma was analyzed for drug concentration.

### Maximum tolerated dose (MTD) and biological safety

Cpt/Epi@ins-FDC at the total drug doses of 30, 35, or 40 mg kg^-1^ body weight (4.05, 4.73, 5.41 mg kg^-1^ Cpt equivalent and 25.95, 30.27, 34.59 mg kg^-1^ Epi equivalent), free Cpt or Epi at the doses of 5, 10, or 15 mg kg^-1^ were i.v. injected to mice. Body weight changes were monitored for two weeks. The highest dose at which no animal mortality and no more than 15% body weight loss was defined as the MTD. Hematology examinations were performed post MTD injection using standard procedures as previously reported 57.

### *In vivo* therapeutic efficacy

The mouse tumor models: U87MG-LUC intracranial glioblastoma, lung metastasized HepG2-LUC, and subcutaneous transplanted MCF7-MDR breast cancer were established according to previous report 55, 58. The initial size of the MCF7-MDR tumor is 50 mm^3^ at day 30 as measured by a fine caliper. For U87MG-LUC glioma and HepG2-LUC lung-metastasized tumor models that grow *in situ* and cannot be measured by a caliper, *in vivo* imaging of tumors were performed on IVIS Spectrum Imaging System to monitor the relative growth rate of tumor according to the intensity and range of LUC fluorescence. The drugs were administrated at day 7 for U87MG-LUC glioma and day 15 for HepG2-LUC lung-metastasized cancer from the first time the tumors can be detected. For U87MG-LUC tumor model, mice were administered with a total drug dose of 3 mg kg^-1^ Cpt/Epi@ins-FDC, Cpt@ins-FDC, Epi@ins-FDC, free Cpt and Epi (the equivalent amount of Cpt and Epi was 0.41 and 2.59 mg kg^-1^) every 3 days for 3 times. For HepG2-LUC and MCF7-MDR tumor model, mice were administrated with a single MTD dose of Cpt/Epi@ins-FDC, Cpt@ins-FDC and Epi@ins-FDC (4.73 mg kg^-1^ Cpt equivalent and 30.27 mg kg^-1^ Epi equivalent), free Cpt and Epi (10 mg kg^-1^), respectively. Tumor images were taken on IVIS Spectrum Imaging System (λex/λem = 365 nm/500 nm) or calculated as tumor volume (V) = L×W^2^/2, where L and W were the length and width of the tumor, respectively. TUNEL staining was performed to measure the apoptosis. Histology examinations of major organs were performed by H&E staining.

### Statistical Analysis

All statistical analyses and comparisons were performed using GraphPad Prism 7.0 software (Graphpad Software Inc., La Jolla, California) and SPSS 17.0 software program (IBM, USA). The statistical significance was assessed via multiple t tests or one-way ANOVA and was defined as *p < 0.05, **p < 0.01, ***p < 0.001,****p < 0.0001. Data were expressed as means ± standard deviations.

## Supplementary Material

Supplementary materials and methods, figures, and tables.
