# Supplementary material for: Re-engineering the inner surface of ferritin nanocage enables dual drug payloads for synergistic tumor therapy
Source: Theranostics. 2022 Jan 24;12(4):1800–15. doi: 10.7150/thno.68459 (PMC8825595; doi:10.7150/thno.68459)
Supplement: Supplementary file 1 — Supplementary materials and methods, figures, and tables. [file thnov12p1800s1.pdf]

Supporting Information

Re-engineering the inner surface of ferritin nanocage enables dual drug payloads for synergistic tumor therapy

Zhuoran Wang[1#], Yue Zhao[1,2#], Shuai Zhang[1,2], Xuehui Chen[1], Guoming Sun[4], Baoli Zhang[1,2], Bing Jiang[3], Yili Yang[5], Xiyun Yan[1,2,3*], Kelong Fan[1,2,3*]

1. CAS Engineering Laboratory for Nanozyme, Key Laboratory of Protein and Peptide, Pharmaceutical Institute of Biophysics, Chinese Academy of Sciences, Beijing, 100101, P. R. China.
2. University of Chinese Academy of Sciences, Beijing, 101408, P. R. China.
3. Nanozyme Medical Center, School of Basic Medical Sciences, Zhengzhou University, Zhengzhou, 450001, P. R. China.
4. Nanjing Nanozyme Tech Co., Ltd., Nanjing, 211500, P. R. China.
5. China Regional Research Center, International Center for Genetic Engineering and Biotechnology, Taizhou, 225312, P. R. China.

[#] These authors contributed equally to this article.

Corresponding authors: Xiyun Yan and Kelong Fan; CAS Engineering Laboratory for Nanozyme, Key Laboratory of Protein and Peptide, Pharmaceutical Institute of Biophysics, Chinese Academy of Sciences, Beijing, 100101, P. R. China. E-mail: yanxy@ibp.ac.cn, fankelong@ibp.ac.cn; Tel: +8601064888584 (Xiyun Yan), +8601064888280 (Kelong Fan).

Supplementary materials and methods

Materials

Peptides were synthesized by Taopu Biology Technology Co. Ltd. (Shanghai, China). Free drugs were obtained from Dalian Meilun Pharm Co. Ltd. (Dalian, China). Dulbecco's modified Eagle's medium (DMEM), RPMI-1640 media, and fetal bovine serum (FBS) were purchased from Gibco (BRL, MD, USA). All other chemicals were of analytical grade.

Preparation of drug-loaded nanocarriers

The gene encoding peptides P6, P13 and P22 were constructed according to the amino acid sequence (AVFAFA, AAVVVFAFAFAFA, and AAAAAVVVVVFAFAFAFAFAFA). The gene sequence encoding HFn was obtained from NCBI (GenBank: DI342556.1 or LG067594.1). The plasmid of ins-FDC nanocarriers were constructed by linking the sequence of P6, P13 and P22 to the C-terminus of the HFn through a flexible amino acid sequence GGSG. As such, the peptide sequences are designed to be located in the inner cavity [1]. The recombinant plasmid ins-FDC-pET-22b (+) was subsequently constructed and transformed into *E. coli.* Transetta (*DE3*) (TransGen Biotech). The transformed *E. coli* cells were cultured in LB medium with 100 mg L^{-1} ampicillin overnight. Then, ins-FDC protein nanocages were expressed by adding 1 mM isopropyl- β -D-thiogalactoside (IPTG, Sigma), then the *E. coli* cells were cultured for 12 h at 30 °C. *E. coli.* cells were collected by centrifugation at 3,000 rpm for 20 min and the pellets were resuspended in Tris buffer (50 mM Tris-HCl, pH 7.4). Resuspended *E. coli.* cells were sonicated on ice and centrifuged at 12,000 rpm for 30 min. The supernatant was heat treated at 80 °C for 15 min to denature and separate most ins-FDC proteins. After centrifugating at 12,000 rpm for 30 min, the proteins were collected from the supernatant and filtrated by 0.22 μm filter. Finally, ins-FDC protein was purified by size exclusion chromatography (SEC) (Amersham Pharmacia Biotech) on a superdexTM 200pg column (GE Healthcare) followed by anion-exchange chromatography on Q-Sepharose Fast

Flow (GE Healthcare). The purification process of proteins was monitored by sodium dodecyl sulfate polyacrylamide gel electrophoresis (SDS-PAGE).

The Cpt and Epi-loaded ins-FDC (Cpt/Epi@ins-FDC) was prepared via a simple urea-thermal incubation method. ins-FDCs were dissolved in 8 M urea at 1 mg mL^{-1} and gently stirred for 30 min at room temperature to ensure fully denaturation and dissolution. Then free Cpt was added into the solution at 1 mg mL^{-1}. After incubation for 30 min at room temperature in the dark, the mixture was transferred into a dialysis bag (molecular weight cut-off 3500 Da, Thermofisher Scientific) and dialyzed against a series of stepwise gradients of urea (7, 5, 3, 2, 1, and 0 M, each for 4 h) in PBS buffer containing Cpt at 1 mg mL^{-1} to refold ins-FDCs protein shell. The resulting solution was then dialyzed against PBS buffer overnight to remove the free Cpt. Free Epi were then added into the Cpt@ins-FDC solution at 1 mg mL^{-1}. After incubation for 4 h at 60 °C in the dark to encapsulate Epi into the inner cavity through the natural drug entry channel, the mixture was transferred into a dialysis bag (molecular weight cut-off 3500 Da, Thermofisher Scientific) and dialyzed against PBS buffer (pH 7.4) for 24 h to remove free drugs.

Cpt@ins-FDC and Epi@ins-FDC were prepared as controls. Cpt/Epi@HFn, Cpt @HFn and Cpt/Epi@HFn were prepared with the same method as above. To verify the spectral properties of the ins-FDC nanocarrier, 5-Fluorouracil/Oxaliplatin@ins-FDC, 5-Fluorouracil/Gemcitabine@ins-FDC, Docetaxel/Gemcitabine@ins-FDC, Docetaxel/Epirubicin@ins-FDC, and Temozolomide/Irinotecan@ins-FDC were prepared by the same procedure as showed above.

Characterizations

The HFn, ins-FDC, and final prepared drug-loaded ins-FDC (Cpt/Epi@ins-FDC, Cpt@ins-FDC and Epi@ins-FDC) were analyzed on a Superdex 200 10/300 GL column (GE Healthcare) connected to a SEC system, using the in-line UV detection at 280 nm (HFn protein cage), 365 nm (Cpt) and 480 nm (Epi) to demonstrate the self-assambly of ins-FDC

and successful loading of drugs. The Cpt and Epi concentration was determined by measuring the absorbance at 365/480 nm and the concentration of ins-FDC was determined in triplicate by a bicinchoninic acid assay (BCA assay) kit (Sigma) using bovine serum albumin as the standard.

The drug loading of 5-Fluorouracil/Oxaliplatin@ins-FDC, 5-Fluorouracil/Gemcitabine@ins-FDC, Docetaxel/Gemcitabine@ins-FDC, Docetaxel/Epirubicin@ins-FDC, and Temozolomide/Irinotecan@ins-FDC were ensured by UV–vis spectra detection at 240 nm (Oxaliplatin), 265 nm (5-Fluorouracil), 260 nm(Gemcitabine), 240 nm (Docetaxel), 480 nm (Epirubicin), 370 nm (Irinotecan) and 300 nm (Temozolomide).

The particle size of nanocarriers was measured on the Malvern Zetasizer NanoZS by dynamic laser scattering (DLS). The system was operated at a scattering angle of 90° and a laser wavelength of 633 nm at 25 °C and the concentration of the samples used was 0.25 mg mL^{-1} in PBS buffer. Samples were filtered before detection. Dynamics 7.1.8 software was used to analyze the data. For transmission electron microscopy (TEM) characterization, the copper TEM grid (Ted Pella Inc.) was plasma glow-discharged for 90 s to create a hydrophilic surface on the carbon surface. Nanocarrier solution (5 μL) was absorbed onto the freshly plasmadischarged carbon membrane for 1 min and then blotted with a filter paper to remove excess solution. The grid was examined with JEOL 2000FX at 120 kV. To investigate the secondary structure of ins-FDC nanocarriers, far-UV circular dichroism (CD) analysis was performed. The circular dichroism spectrum was obtained at 190 nm to 260 nm in a 0.1 cm quartz cuvette using a Jasco J-810 spectropolarimeter (Jasco, Japan). Samples (0.5 mg mL^{-1}) were first desalted with 20 mM PB (pH 7.4) before detection, and the background spectrum of the buffer was recorded and subtracted from subsequent readings. The software package Spectra Manager (Jasco, Japan) was used for data collection and analysis.

***In vitro* drug release and stability study**

The release profiles of Cpt and Epi from Cpt/Epi@ins-FDC were assessed in PBS at pH values of 7.4 and 5.0. For each release study, 10 mg of Cpt/Epi@ins-FDC was suspended in 2 mL of PBS solution, and the solution was put into a dialysis tube that was directly immersed into 30 mL of buffer solution and maintained at 37 °C. The concentrations of the released drugs were calculated by the measurement of the fluorescence intensity (λex = 365 nm for Cpt and λex = 480 nm for Epi) with reference to the standard curve. After each sampling, the buffer solution was refreshed immediately. Cpt/Epi@ins-FDC was incubated with PBS or normal mouse serum for stability evaluation of Cpt and Epi.

Cell culture

Three types of human tumor cell lines, including U87MG, HepG2 and MCF7-MDR, and human aortic smooth muscle cells (hASMC) were cultured in Dulbecco's modified Eagle's medium (DMEM) that contains 10% (v/v) fetal bovine serum, 100 IU mL^{-1} penicillin and 100 μg mL^{-1} streptomysin at 37 °C in a humidified 5% CO_2-95% air atmosphere.

Cell-binding assays of Cpt/Epi@ins-FDC

Confocal laser scanning microscope (CLSM). To confirm the receptor mediated targeting of Cpt/Epi@ins-FDC interacted with CD71 for internalization improvement, U87MG cells were cultured in 35 mm confocal dishes (Corning). Once cells reached 90% confluency, Cpt/Epi@ins-FDC (10 μM) was added into U87MG cells for 2 h after pre-incubated with or without anti-CD71 monoclonal antibody. Cells were then washed three times with PBS and fixed in 4% cold formaldehyde in PBS for 10 min at room temperature. After washing with PBS, the cell nuclei were further stained by propidium iodide (PI). Fluorescence intensity of Cpt was quantified by Image J software.

FACS. ins-FDC and HFn were labeled with the fluorescent dye Cy5 by the following procedures. The Cy5-NHS ester (GE Healthcare) was dissolved in DMSO (Sigma-Aldrich) and added to ins-FDC or HFn solution (0.1 M $NaHCO_3$, pH 8.5), at a dye to ins-FDC or HFn molar ratio of 10 : 1. The mixture was gently stirred overnight at 4 °C in the dark and then

purified by dialysis (molecular weight cut-off 6,000) to remove free dyes. The concentration of labeled Cy5 was determined by measuring the absorbance at 650 nm and the concentrations of ins-FDC and HFn were determined using a bicinchoninic acid assay (BCA assay) kit (Sigma). The Cy5-ins-FDC was further loaded with Cpt or Epi by the same procedure described previously.

To perform the binding analysis, 100 μL detached U87MG cell suspensions (2.5×10^6 cells mL^{-1}) were incubated with 10 μM of Cy5-conjugated Cpt/Epi@ins-FDC, Cpt@ins-FDC, Epi@ins-FDC, ins-FDC, HFn and PBS for 45 min at 4°C. Cells washed with PBS for three times were then analyzed by a FACSCalibur flow cytometry system. Competitive binding assay was further performed by using FACS. U87MG cells were incubated with Cy5-labeled HFn in the presence of increasing concentrations of unlabeled Cpt/Epi@ins-FDC, ins-FDC or HFn. The fluorescence of Cy5 was evaluated to quantitatively compare the binding activity of Cpt/Epi@ins-FDC, ins-FDC and HFn to CD71.

Intracellular trafficking of Cpt/Epi@ins-FDC

To visualize the intracellular uptake and drug release of Cpt/Epi@ins-FDC, U87MG cells were cultured in 35 mm Petri dishes (1×10^5 cells/well). Following incubation with 10 μM Cpt/Epi@ins-FDC at 37 °C for 0.25, 0.5, 2, 6 and 24 h, the cells were washed with cold PBS, and fixed with 4% formaldehyde for 20 min at room temperature. Lysosomes were stained with Lysosomal Associated Membrane Protein 1 (LAMP1), and the cell nuclei were further stained with PI according to the standard protocol provided by the supplier. Then cells were washed with ice-cold PBS. The intracellular trafficking behavior of Cpt/Epi@ins-FDC was visualized under a confocal laser scanning microscope (CLSM, Carl Zeiss LSM 700).

***In vitro* cytotoxicity**

The cytotoxicities of Cpt/Epi@ins-FDC P6, Cpt/Epi@ins-FDC P13, and Cpt/Epi@ins-FDC P22 on U87MG cells were assessed by cell counting kit-8 (CCK8) assay. Cells were seeded in 96-well plates with 7,000 cells per well in 100 μL of DMEM medium and incubated at 37 °C

in a 5% CO_2 atmosphere for 24 h, followed by removing culture medium and then adding Cpt/Epi@ins-FDC (200 μL in DMEM medium) with different concentrations. The cells were subjected to CCK8 assay after being incubated for another 48 h. After 2 h of reaction incubation, the UV absorbance of 450 nm was measured using a Varioskan Flash Spectral Scanning Multimode Reader (ThermoFisher Scientific). The half-maximal inhibitory concentration (IC50) was calculated based on the protein concentration as previously reported [2] using the GraphPad Prism 7.0 software (GraphPad Software, CA, USA). The *in vitro* cytotoxicities of Cpt/Epi@ins-FDC, Cpt@ins-FDC, Epi@ins-FDC，free Cpt, free Epi and ins-FDC on three types of cancer cell lines (U87MG, HepG2 and MCF7-MDR) and human aortic smooth muscle cells (hASMC) were compared. The cytotoxicities of 5-Fluorouracil/Oxaliplatin@ins-FDC, 5-Fluorouracil/Gemcitabine@ins-FDC, Docetaxel/Gemcitabine@ins-FDC, Docetaxel/Epirubicin@ins-FDC, and Temozolomide/Irinotecan@ins-FDC were detected using the same method.

The inhibitory concentration (ICx) values were determined using Origin 8.0 (OriginLab, Northampton, MA) according to the fitted data. The Combination Index (CI) was measured according to the Chou and Talalay's method [3]. To distinguish synergistic, additive, or antagonistic cytotoxic effects, the following equation was used:

$$CIx = D_1/Dx_1 + D_2/Dx_2 \qquad \text{Eq. (1)}$$

D_1 and D_2 are the concentrations of drug 1 and drug 2, respectively, that function in combination to achieve a specified drug effect (e.g., 50% inhibition of cell viability). Dx_1 and Dx_2 are the doses for single drugs to achieve the same effect. CI > 1 represents antagonism, CI =1 represents additive and CI < 1 represents synergism. In this work, IC50 (inhibitory concentration to produce 50% cell death) was applied.

Cell colony assay

To detect the enhanced tumor therapy of Cpt/Epi@ins-FDC in a longer period, cell colony formation assay was also conducted. In brief, 2×10^3 of U87MG cells were seeded into 6-well

plates in all groups, following incubation with 10 μM drug-loaded ins-FDC nanocarriers at 37 °C for 24 h. Then the cells were washed with cold PBS, and fresh culture medium was added. Once macroscopic colonies formed after cultivating for 10 days, crystal violet solution was applied to investigate the amount of cell colonies.

***In vitro* therapeutic effect of Cpt/Epi@ins-FDC**

In order to inspect the therapeutic effect of Cpt/Epi@ins-FDC *in vitro*, 3D cell spheroids were formed by seeding 2×10^3 MCF7-MDR cells into ultra-low attach attachment round bottom 96-well plates (Corning, 7007, America). After cell spheroid formation, different treatments including 20 μM of Cpt/Epi@ins-FDC, Cpt@ins-FDC, Epi@ins-FDC， free Cpt, free Epi and PBS were administered on day 0, respectively. The images and diameters of the cell spheroids were recorded and analyzed using Nikon NIS-Elements every other day. The volume of the spheroids was calculated according to formula:

$$V = 4/3 \times \pi \times (d/2)^2 \qquad \text{Eq. (2)}$$

***In vivo* study**

Animal Models. All animal studies were performed following the ethics protocol approved by the Institutional Animal Care and Use Committee at the Institute of Biophysics, Chinese Academy of Sciences (approval number: SYXK2019010). Female BALB/c nude mice (6-week-old) were obtained from Vital River Laboratories (Beijing). Mice were housed under standard conditions with free access to sterile food and water.

To establish the U87MG-LUC intracranial orthotopic glioblastoma mice model, mice were anesthetized using 2.0% isoflurane and then positioned in a stereotactic instrument. The top of the animal's head was cleaned with 75% ethanol and a linear skin incision was made over the bregma. A 27G needle was then used to drill a burrhole into the skull 0.5 mm anterior and 2 mm lateral to the bregma. A 10 μL gastight syringe (Hamilton) was then used to inject 10 μL of the luciferase-transfected human glioblastoma cell line U87MG-LUC cell suspension (1×10^6 cells in PBS) in the striatum at a depth of 2.5 mm from the dural surface. The injection

was done slowly over 10 min. Then the skin was closed with sutures. For whole-body imaging, each tumor-bearing mouse was injected with drug samples via tail vain, the luciferase-catalyzed bioluminescence of tumor and signals of Cpt fluorescence were then visualized with a IVIS Spectrum Imaging System (Caliper, USA) according to previous report [4].

For lung metastases mouse model therapeutic assessment, female BALB/c nude mice of 6-week-old were i.v. injected with 2×10^6 HepG2-LUC tumor cells. Two weeks later, tumors could be imaged by detecting the signal of luciferase using IVIS Spectrum Imaging System.

For subcutaneous in situ transplanted drug-resistant breast tumor model, MCF7-MDR tumor cells (1×10^7) were implanted into the mice mammary fat pads. After 30 days, the tumor volume reached to 50 mm^3.

***In vivo* biodistribution and pharmacokinetics study.** For biodistribution study, after the glioma mice model were established, Cpt/Epi@ins-FDC, Cpt@ins-FDC and free Cpt were injected with a dose of 3 mg kg^{-1} Cpt equivalents through the tail vein (n = 3 for each group). After 1, 4, and 24 h of injection, the tumors as well as major organs were collected from the mice after euthanasia and subjected for ex vivo imaging. The near-infrared (near-IR) optical images were taken on IVIS Spectrum Imaging System and the fluorescence intensities were analyzed by Living Image Software.

To determine the pharmacokinetics, Cpt/Epi@ins-FDC in equivalent drug doses of 1.35 mg kg^{-1} Cpt and 8.65 mg kg^{-1} Epi, Cpt@ins-FDC (1.35 mg kg^{-1}, Cpt equivalent), Epi@ins-FDC (8.65 mg kg^{-1}, Epi equivalent), free Cpt (1.35 mg kg^{-1}), and free Epi (8.65 mg kg^{-1}), were i.v. injected into BALB/c nude mice (n = 5 for each group). At various times after injection blood was collected from the tail vein and the plasma was separated and analyzed for drug concentration by the following procedure. Briefly, the obtained plasma samples (10 μL) were incubated with 490 μL of acidified isopropanol (0.75 M HCl in isopropanol) overnight at −20 °C in the dark to extract free drugs. The mixture was then centrifuged at

15,000 × g for 20 min and the supernatant was loaded onto a 96-well plate (Corning). Cpt concentration was determined by measuring the fluorescence at 365 nm excitation, using a Varioskan Flash Spectral Scanning Multimode Reader (ThermoFisher Scientific). The fluorescence of blood samples from untreated mice was determined to correct for nonspecific background. Then the drug concentration of blood samples was quantified by comparing to a linear standard curve that was created between the fluorescence values and standard free drug concentration from 1 to 3,000 nM.

Maximum tolerated dose (MTD). 6 week old healthy female SPF BALB/c mice were i.v. injected with Cpt/Epi@ins-FDC at the doses of 30, 35, or 40 mg kg^{-1} body weight (4.05, 4.73, 5.41 mg kg^{-1} Cpt equivalent and 25.95, 30.27, 34.59 mg kg^{-1} Epi equivalent), free Cpt at the doses of 5, 10, or 15 mg kg^{-1} or free Epi at the doses of 5, 10, or 15 mg kg^{-1} (n = 3 for each injection). Body weight changes and survival of mice were monitored every day for two weeks. The mice that lost over 15% of their pre-treatment body weights were euthanized. The highest dose at which no animal mortality and no more than 15% body weight loss was defined as the MTD.

Biological safety. To evaluate the *in vivo* safety of Cpt/Epi@ins-FDC, the hematology examination were performed, the blood samples were collected via the retro-orbital and analyzed by Automatic Biochemical Analyzer to obtain clinical blood chemistry parameters and hematological parameters as follows: Alanine aminotransferase (ALT) and aspartate aminotransferase (AST) are markers for hepatic function. Creatinine (CREA) and blood urea nitrogen (UREA)are markers for kidney function. Creatine kinase isoenzymes (CK-MB) and lactate dehydrogenase (LDH) are markers for heart function.

***In vivo* therapeutic efficacy.** *In vivo antitumor efficacy to U87MG-LUC tumor model*: After being weighed and randomly divided into 6 groups, the mice were i.v. injected with a total drug dose of 3 mg kg^{-1} Cpt/Epi@ins-FDC (the amount of Cpt and Epi was 0.41 and 2.59 mg kg^{-1}), Cpt@ins-FDC and free Cpt in equivalent drug doses of 0.41 mg kg^{-1} Cpt, Epi@ins-FDC

and free Epi in equivalent drug doses of 2.59 mg kg^{-1} Epi every 3 days for 3 times, respectively (n = 5-6 for each group). Images were taken on IVIS Spectrum Imaging System every 3 days post injection. Body weight changes and survival of mice were measured during experimental period. For TUNEL apoptosis staining, the fixed tumor sections were stained by the In Situ Cell Death Detection Kit (Roche Applied Science) according to the manufacturer's protocol. DAPI was used for nuclear counterstaining. The stained tumor slides were detected by fluorescence microscope (IX71, Olympus). The major tissues were fixed with formalin. Histology detection was performed by hematoxylin and eosin (H&E) staining for morphology observation using standard procedures as previously reported [5]. The images were captured with a Nikon Eclipse 90i microscope.

In vivo antitumor efficacy to HepG2-LUC tumor model: The mice were weighed, randomly divided into 6 groups, and i.v. administrated with a single MTD dose of Cpt/Epi@ins-FDC (4.73 mg kg^{-1} Cpt equivalent and 30.27 mg kg^{-1} Epi equivalent), free Cpt (10 mg kg^{-1}), free Epi (10 mg kg^{-1}), Cpt@ins-FDC (4.73 mg kg^{-1}, Cpt equivalent), and Epi@ins-FDC (30.27 mg kg^{-1}, Epi equivalent), respectively (n = 5 for each group). The *in vivo* imaging of tumor and body weight of the mice were measured every 3 days. At day 30, the lungs were collected from the mice after euthanasia, washed by saline thrice and then fixed in 10% neutral buffered formalin (NBF). For H&E staining, formalin-fixed lungs were embedded in paraffin blocks and visualized by optical microscope (DM5500B, Leica).

In vivo antitumor efficacy to MCF7-MDR tumor model: When the tumor volume reached to 50 mm^3 at day 30, the mice were weighed, randomly divided into 6 groups, and i.v. injected with Cpt/Epi@ins-FDC, Cpt@ins-FDC, Epi@ins-FDC， free Cpt, free Epi at their MTDs as above, and PBS at the equivalent volume, respectively (n = 5 for each group). Body weight changes and survival of mice were measured every 3 days. Tumor size was monitored by a fine caliper and the tumor volume (V) was calculated as $V=L\times W^2/2$, where L and W were the length and width of the tumor, respectively.

Statistical Analysis

All statistical analyses and comparisons were performed using GraphPad Prism 7.0 software (Graphpad Software Inc., La Jolla, California) and SPSS 17.0 software program (IBM, USA). The statistical significance was assessed via multiple t tests or one-way ANOVA and was defined as $^{*}p < 0.05$, $^{**}p < 0.01$, $^{***}p < 0.001$,$^{****}p < 0.0001$. Values are calculated with means and standard deviations.

Reference

[S1] Jin Y, He J, Fan K, Yan X. Ferritin variants: inspirations for rationally designing protein nanocarriers. Nanoscale. 2019; 11: 12449-59.

[S2] Wang Z, He Q, Zhao W, Luo J, Gao W. Tumor-homing, pH- and ultrasound-responsive polypeptide-doxorubicin nanoconjugates overcome doxorubicin resistance in cancer therapy. J Control Release. 2017; 264: 66-75.

[S3] Chou T. Theoretical basis, experimental design, and computerized simulation of synergism and antagonism in drug combination studies. Pharmacol Rev. 2006; 58: 621-81.

[S4] Fan K, Jia X, Zhou M, Wang K, Conde J, He J, et al. Ferritin nanocarrier traverses the blood brain barrier and kills glioma. ACS Nano. 2018; 12: 4105-15.

[S5] Wang Z, Guo J, Sun J, Liang P, Wie Y, Deng X, et al. Thermoresponsive and protease-cleavable interferon-polypeptide conjugates with spatiotemporally programmed two-step release kinetics for tumor therapy. Adv Sci (Weinh). 2019; 6: 1900586.

Supplementary figures and tables

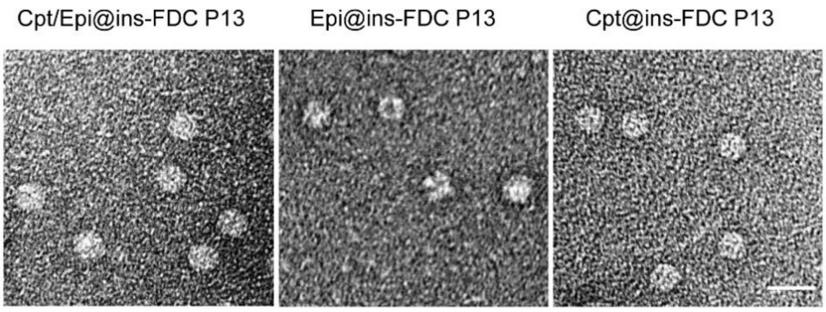

Figure S1. TEM images of the drug-loaded ins-FDC P13 nanocarriers. Scale bar = 20 nm.

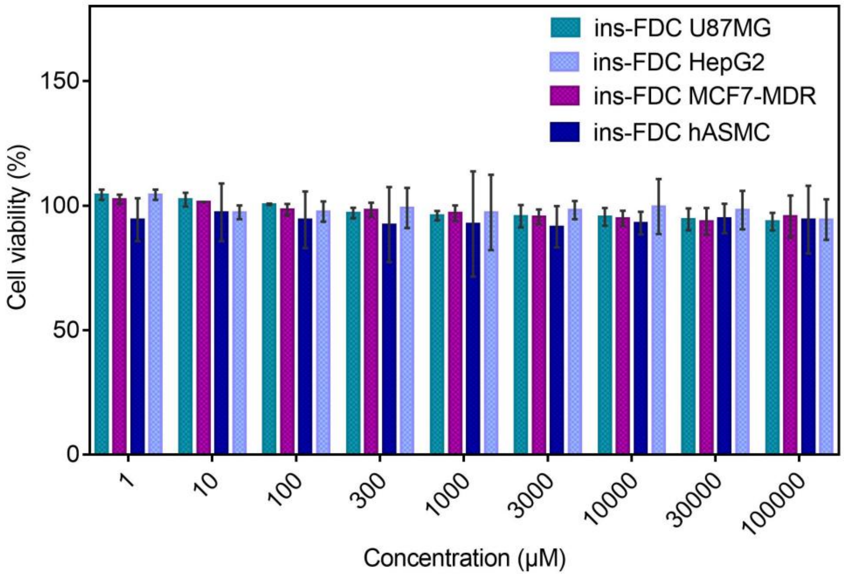

Figure S2. Cytotoxicity of ins-FDC at different concentrations in U87MG, HepG2, MCF7-MDR and hASMC cell lines. The data represent the mean ± SD from 3 replicates for each run.

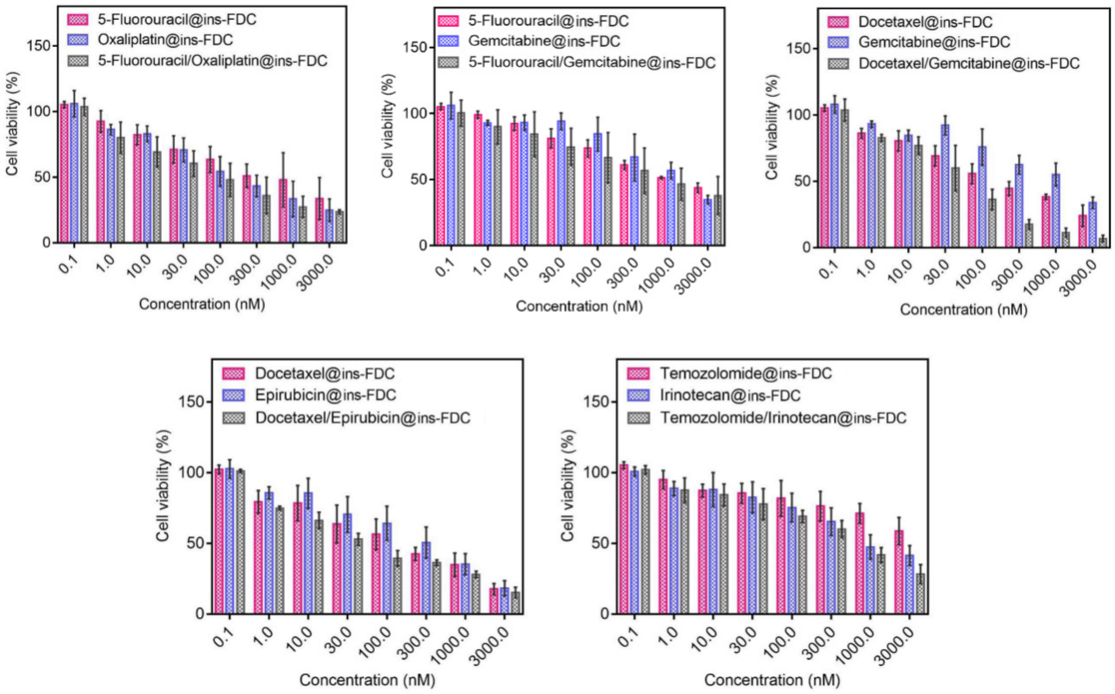

Figure S3. Cytotoxicity assays of ins-FDC after loading different hydrophobic and hydrophilic drugs. Data are shown as means ± SD (n = 3).

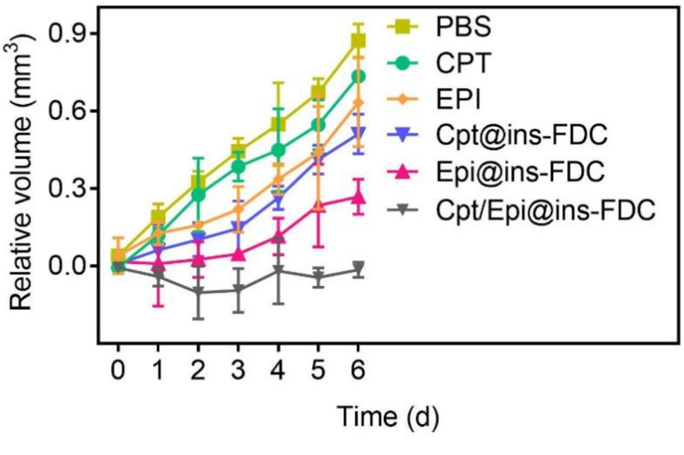

Figure S4. Dynamic volume of MCF7-MDR cell spheroids. Data are shown as means ± SD (n = 3).

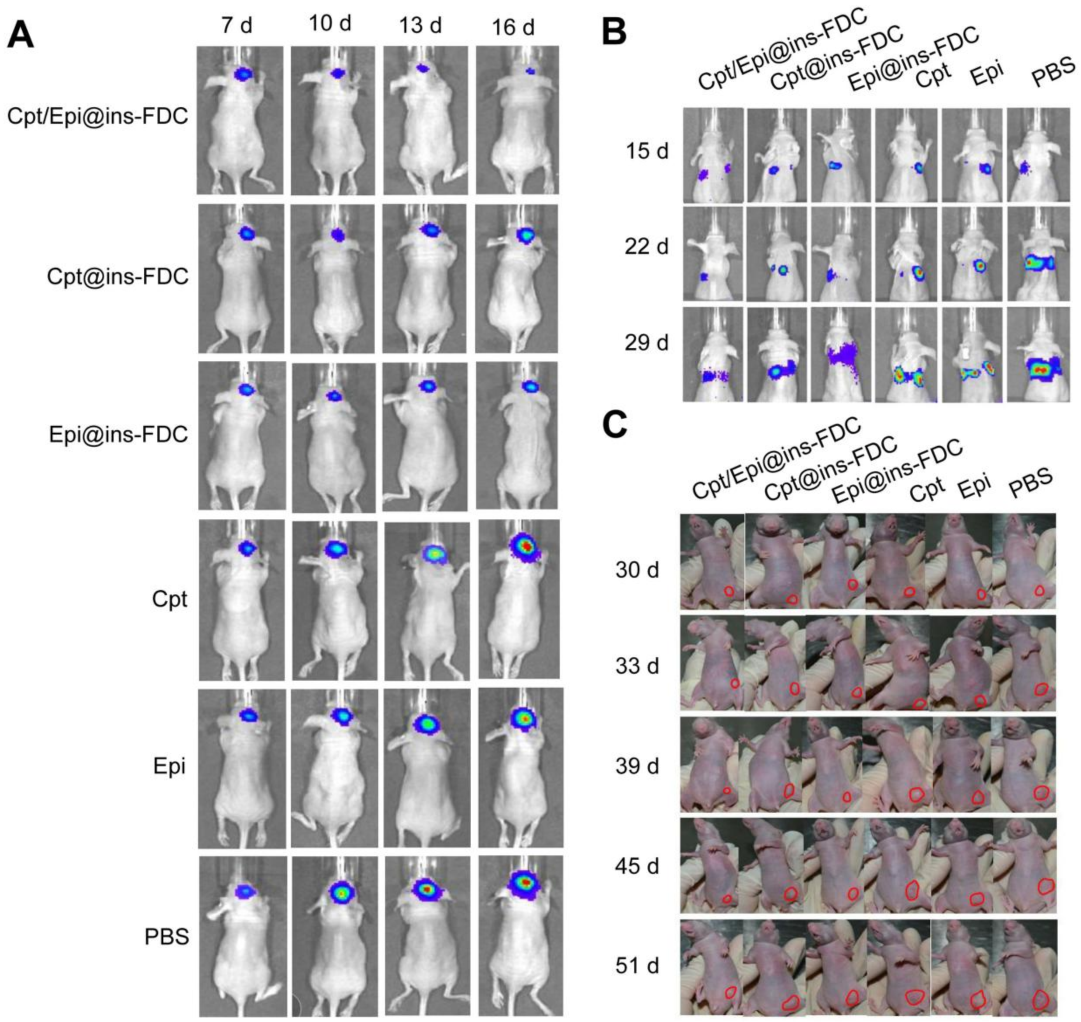

Figure S5. *In vivo* bioluminescence images of U87MG-LUC glioma (A), HepG2-LUC (B) and digital pictures of MCF7-MDR bearing mice (C) that were i.v. injected with different formulations.

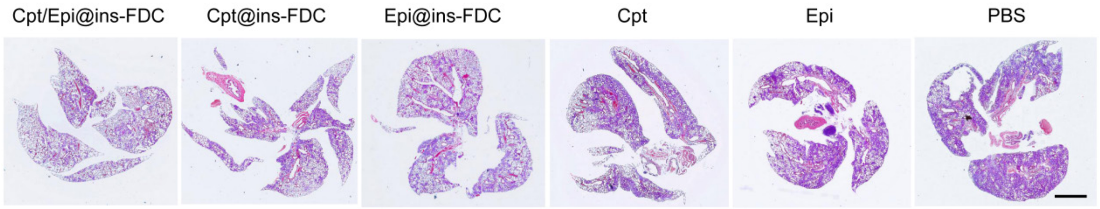

Figure S6. H&E staining of paraffin-embedded lung slices with tumor nodules from Cpt/Epi@ins-FDC administrated mice. Scale bar = 2 mm.

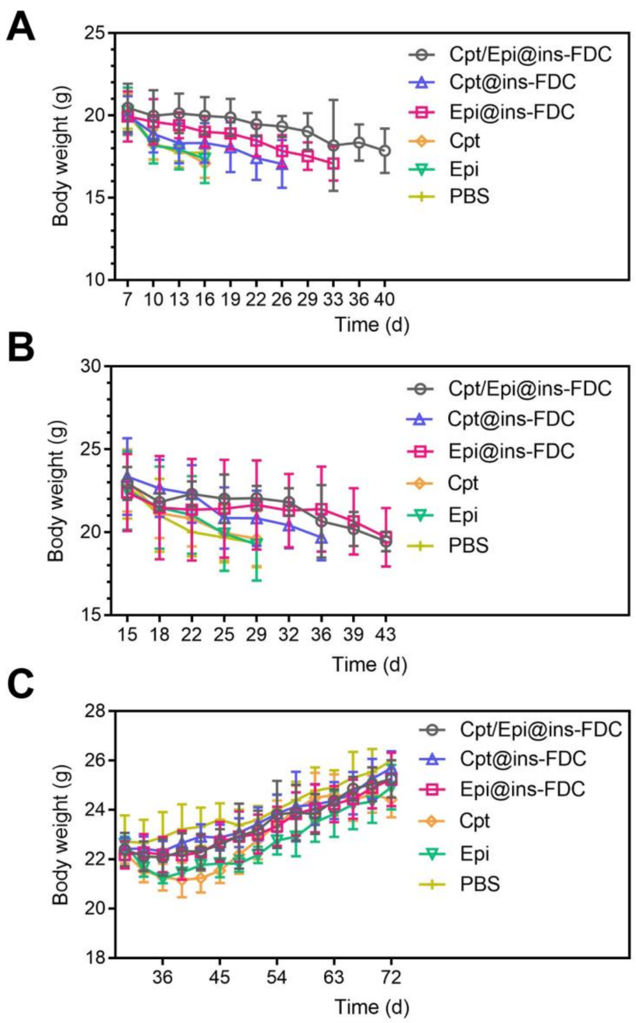

Figure S7. Changes in body weight of U87MG-LUC glioma (A), HepG2-LUC (B) and MCF7-MDR (C) tumor bearing mice with different treatments. Data are shown as means ± SD (*n* = 5).

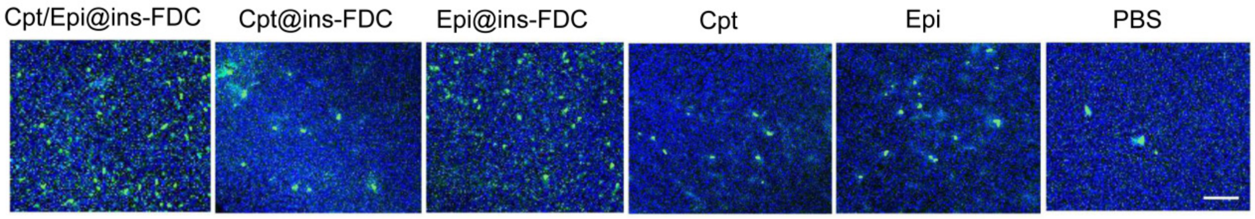

Figure S8. Representative photomicrographs of TUNEL assay.

Table S1. The number of drugs loaded in ins-FDC P6, P13, and P22.

	Hydrophobic amino acid number	Hydrophilic drug number	Hydrophobic drug number	Total drug number
Cpt/Epi@ins-FDC P6	6	162	8	170
Cpt/Epi@ins-FDC P13	13	150	39	189
Cpt/Epi@ins-FDC P22	22	82	45	127

Table S2. IC50 values for Cpt/Epi@ins-FDCs with different PDRs to U87MG cells. Data are shown as means ± SD (*n* =3).

**U87 cell line**	**IC50 (μM)**
Cpt/Epi@ins-FDC P6	0.049 ± 0.0087
Cpt/Epi@ins-FDC P13	0.031 ± 0.011
Cpt/Epi@ins-FDC P22	0.066 ± 0.013

Table S3. IC50 values for Cpt/Epi@ins-FDC, ins-FDC and HFn in the analysis of the competitive binding curves.

**U87 cell line**	**IC50 (μM)**
Cpt/Epi@ins-FDC	0.50 ± 0.050
ins-FDC	0.51 ± 0.10
HFn	0.51 ± 0.080

Table S4. IC50 values of Cpt/Epi@ins-FDC, Cpt@ins-FDC, Epi@ins-FDC, Cpt, Epi and ins-FDC to U87MG, HepG2, MCF7-MDR and hASMC cells. Data are shown as means ± SD (*n* =3).

	**IC50 (μM)**			
	U87MG	HepG2	MCF7-MDR	hASMC
Cpt/Epi@ins-FDC	0.033 ± 0.017	0.031 ± 0.011	0.14 ± 0.032	0.094 ± 0.0018
Cpt@ins-FDC	0.46 ± 0.20	0.26 ± 0.087	1.54 ± 0.055	0.88 ± 0.022
Epi@ins-FDC	0.096 ± 0.032	0.12 ± 0.033	0.31 ± 0.035	0.23 ± 0.0021
Cpt	8.38 ± 0.058	7.19 ± 0.11	1204 ± 18.32	15.07 ± 0.14
Epi	3.73 ± 0.093	4.87 ± 0.12	467.1 ± 64.11	11.34 ± 0.11
ins-FDC	-	-	-	-

Table S5. IC50 values of ins-FDC after loading different hydrophobic and hydrophilic drugs. Data are shown as means ± SD (*n* = 3).

			**IC50 (μM)**		
Hydrophobic / Hydrophilic drug	5-Fluorouracil / Oxaliplatin	5-Fluorouracil / Gemcitabine	Docetaxel / Gemcitabine	Docetaxel / Epirubicin	Temozolomide / Irinotecan
Representative cell line	Hepatocellular carcinoma cell HepG2	Hepatocellular carcinoma cell HepG2	Hepatocellular carcinoma cell HepG2	Drug resistant breast cancer cell MCF7-MDR	Glioma cell U87MG
Hydrophobic drug@ins-FDC	0.57 ± 0.049	1.18 ± 0.057	0.21 ± 0.067	0.15 ± 0.041	5.31 ± 0.12
Hydrophilic drug@ins-FDC	0.19 ± 0.043	1.22 ± 0.071	1.03 ± 0.074	0.25 ± 0.079	1.14 ± 0.085
Hydrophobic / Hydrophilic drug@ins-FDC	0.084 ± 0.037	0.69 ± 0.15	0.045 ± 0.0059	0.048 ± 0.026	0.51 ± 0.054

Table S6. Pharmacokinetic parameters of Cpt/Epi@ins-FDC, Cpt@ins-FDC, Epi@ins-FDC, free Cpt, and free Epi after intravenous injections (*n* = 3).

	Cpt	Epi	Cpt@ins-FDC	Epi@ins-FDC	Cpt/Epi@ins-FDC
$t_{1/2}$ (h)	0.58 ± 0.13	0.70 ± 0.16	6.79 ± 0.30	7.15 ± 0.48	7.20 ± 0.14
AUC (%ID/mL*h)	1.97 ± 0.55	1.87 ± 0.87	164.52 ± 42.66	167.15 ± 24.16	177.95 ± 19.75
CL (L/h/kg)	2.42 ± 0.69	3.20 ± 1.74	0.032 ± 0.008	0.030 ± 0.004	0.026 ± 0.004
Vd (L/kg)	1.84 ± 0.92	3.43 ± 1.11	0.31 ± 0.07	0.31 ± 0.04	0.26 ± 0.04
